# Does imbalance in chest X-ray datasets produce biased deep learning approaches for COVID-19 screening?

**DOI:** 10.1186/s12874-022-01578-w

**Published:** 2022-04-28

**Authors:** Lorena Álvarez-Rodríguez, Joaquim de Moura, Jorge Novo, Marcos Ortega

**Affiliations:** 1 Centro de Investigación CITIC, Universidade da Coruña, Campus de Elviña, A Coruña, 15071 Spain; 2 Grupo VARPA, Instituto de Investigación Biomédica de A Coruña (INIBIC), Universidade da Coruña, A Coruña, 15006 Spain

**Keywords:** CAD system, Chest X-ray, COVID-19 screening, Data analysis, Deep learning

## Abstract

**Background:**

The health crisis resulting from the global COVID-19 pandemic highlighted more than ever the need for rapid, reliable and safe methods of diagnosis and monitoring of respiratory diseases. To study pulmonary involvement in detail, one of the most common resources is the use of different lung imaging modalities (like chest radiography) to explore the possible affected areas.

**Methods:**

The study of patient characteristics like sex and age in pathologies of this type is crucial for gaining knowledge of the disease and for avoiding biases due to the clear scarcity of data when developing representative systems. In this work, we performed an analysis of these factors in chest X-ray images to identify biases. Specifically, 11 imbalance scenarios were defined with female and male COVID-19 patients present in different proportions for the sex analysis, and 6 scenarios where only one specific age range was used for training for the age factor. In each study, 3 different approaches for automatic COVID-19 screening were used: Normal vs COVID-19, Pneumonia vs COVID-19 and Non-COVID-19 vs COVID-19. The study was validated using two public chest X-ray datasets, allowing a reliable analysis to support the clinical decision-making process.

**Results:**

The results for the sex-related analysis indicate this factor slightly affects the system in the Normal VS COVID-19 and Pneumonia VS COVID-19 approaches, although the identified differences are not relevant enough to worsen considerably the system. Regarding the age-related analysis, this factor was observed to be influencing the system in a more consistent way than the sex factor, as it was present in all considered scenarios. However, this worsening does not represent a major factor, as it is not of great magnitude.

**Conclusions:**

Multiple studies have been conducted in other fields in order to determine if certain patient characteristics such as sex or age influenced these deep learning systems. However, to the best of our knowledge, this study has not been done for COVID-19 despite the urgency and lack of COVID-19 chest x-ray images. The presented results evidenced that the proposed methodology and tested approaches allow a robust and reliable analysis to support the clinical decision-making process in this pandemic scenario.

**Supplementary Information:**

The online version contains supplementary material available at (10.1186/s12874-022-01578-w).

## Background

In March 2020, the World Health Organization (WHO) declared the COVID-19 outbreak a pandemic. This highly contagious disease caused by the Severe Acute Respiratory Syndrome Coronavirus 2 (SARS-CoV-2) overwhelmed the healthcare system of many countries, forcing them to take drastic measurements to control the incessant flow of infected patients such as lockdown and curfew, among others health measures. This health crisis resulting from the global COVID-19 pandemic caused more than 346 million confirmed cases and more than 5.5 million deaths worldwide cite[1], highlighting more than ever the necessity of rapid, reliable and safe methods of diagnosing and monitoring respiratory diseases. COVID-19 is a demonstration of the impact that these diseases can have on society, with direct repercussions on public health and the global economy. Due to its particularities, these diseases present a very high transmission rate, as they can be easily transmitted by air. In this context, early detection and assessment of the evolution of patients with these diseases is vital, since many of them in their most severe phases, can lead to symptoms including acute respiratory failure, requiring the use of assisted breathing systems or admission to an intensive care unit (ICU).

**Fig. 1:**
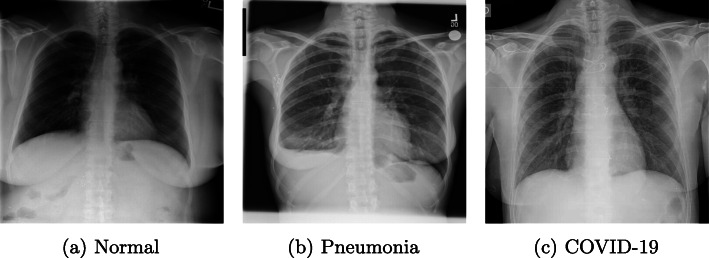
Representative examples of chest X-ray images of normal (patient without pulmonary conditions), patient with pneumonia (others than COVID-19) and patient with COVID-19

Efforts in the deep learning domain have been devoted to improving COVID diagnostics in several fronts, like by combining RT-PCR and pseudo-convolutional machines to characterize virus sequences cite[2]. In order to study lung involvement in detail, one of the most common resources is to use different lung imaging modalities (such as chest X-ray) to explore the possible affected areas. This requires a detailed analysis to identify and characterize the different pathological structures on the chest X-ray image, which should be performed by a professional with many years of experience. In this sense, the need to have a set of computational methodologies that allow detailed analysis of a chest X-ray image for diagnostic purposes is critical, especially in the current pandemic scenario. As reference, Fig. 1 shows 3 representative examples of chest X-ray images for 3 different scenarios: normal (patient without pulmonary conditions), patient with pneumonia (others than COVID-19) and patient with COVID-19.

Given the great relevance of this topic, different authors have developed methodologies to support the diagnosis of COVID-19 using X-ray imaging [3, 4]. As reference, Wang et al. [5] developed an open access customized convolutional neural network (CNN) that detects COVID-19 signs in chest X-ray images. Along with this system, they also provided a public dataset named COVIDx that combines images from the main COVID-19 public datasets. In the work of Hammoudi et al. [6], the author proposed a deep learning system that distinguished bacterial pneumonia from viral pneumonia which could be caused by COVID-19. COVIDX-Net is a framework presented by Hemdan et al. [7] whose purpose is organizing seven different chest X-ray classifiers in order to diagnose COVID-19. In the work of Zhang et al. [8], the authors used Confidence Aware Anomaly Detection (CAAD) models to differentiate viral pneumonia from non-viral pneumonia and non-infected patients. Ozturk et al. [9] designed the DarkCovidNet, a deep learning architecture based on DarkNet, and their work was validated by a radiologist who reviewed heatmaps that showed where their system was identifying anomalies related to COVID-19. Gomes et al. cite[10] created IKONOS, a tool to support diagnosis of COVID-19 by texture analysis of X-ray images. Ismael et al. cite[11] used multiresolution approaches, like Wavelet, Shearlet and Contourlet transforms, for feature extraction for chest X-ray image based COVID-19 detection to prove these traditional methods are still effective. Shelke et al. [12] proposed a methodology that classified chest X-ray into normal, pneumonia, tuberculosis and COVID-19 classes, being able to rate severity. Yoo et al. [13] proposed a methodology based on classification trees that categorized X-ray images between normal and anomalies, and COVID-19 and non-COVID-19, respectively. Ismael et al. cite[14] considered deep feature extraction from pretrained deep networks, fine-tuning of a pretrained CNN model, and end-to-end training of a CNN model to classify chest X-rays into NORMAL and COVID-19 classes. In the work of Li et al. [15], the authors made predictions about a COVID-19 infected patient outcome by using a Siamese convolutional neural network [16] to estimate the disease severity. They used chest X-ray images to prognosticate patient’s intubation or death, which is a useful resource for hospital resources management. De Moura et al. [17] presented 3 complementary approaches based on Dense Convolutional Network architectures specifically designed for the classification of chest X-ray images into normal, pathological and COVID-19. Waheed et al. [18] addressed the lack of COVID-19 chest X-ray and they tried to solve this by developing CovidGAN, a model based on Auxiliary Classifier Generative Adversarial Network that generates synthetic COVID-19 images. In the work of Morís et al. [19], the authors proposed a strategy to improve the performance of COVID-19 screening [20] by using 3 CycleGAN architectures to generate synthetic images from portable chest X-ray devices.

Nowadays, there is no doubt that deep learning methods are useful resources in the field of medical image analysis. However, these methods require a large amount of data for the developed systems to be used in a real scenario. This problem is known as data scarcity and exists even for more researched and common diseases, such as cancer or pneumonia, whose public datasets are scarce and, some of them, unbalanced, containing only certain types of patients. For instance, the Kaggle Pneumonia dataset [21] that was widely used in the development of different systems for automatic COVID-19 screening only contains pediatric chest X-ray images. This problem was commented by Cirillo et al. [22] in their work, as they describe how biased systems produce discriminatory results in the medical field. They focus on the sex and gender factors, as they consider these aspects to affect diseases, risks, treatments, symptoms, etc. In the work of Larrazabal et al. [23], the authors analysed how imbalance related to gender slightly biases deep learning systems when diagnosing some lung pathologies and abnormalities through chest X-ray images, even though observed worsening was not large. In the work of Vidal et al. [24], the authors proposed a methodology that attempts to alleviate this data scarcity problem in the COVID-19 domain by a two-step knowledge transfer to obtain a robust system able to segment lung regions from portable X-ray devices despite the scarcity of samples and lesser quality. However, to date, to the best of our knowledge, no such study, specifically for sex and age, has been performed for COVID-19 despite all the advances, number of articles and studies, the urgency and lack of COVID-19 chest-x ray images.

Therefore, in this work, we performed a comprehensive analysis of sex and age factors in the COVID-19 datasets. As mentioned above, these characteristics might influence the diagnosis of a disease of this type, where there is a clear problem of data scarcity, which may take us away from the goal of having systems that are as representative as possible and gaining more knowledge about the pathology itself. By thoroughly studying these patient characteristics, we made sure to answer the question of whether these factors produce bias in COVID-19 deep learning-based systems. For this purpose, we analyzed 3 different computational approaches for COVID-19 screening using chest X-ray images: (I) Normal vs COVID-19, (II) Pneumonia vs COVID-19 and (III) Non-COVID-19 vs COVID-19. The proposed study was validated using two state-of-the-art datasets publicly available to the scientific community.

This paper is organized as follows: “Methods” section describes the resources and deep learning approaches employed for the analysis of sex and age factors in the COVID-19 datasets; “Results” section presents the obtained results; and finally, “Discussion” and “Conclusions” sections conclude the manuscript, discussing the results and their impact in relation to the state of the art.

## Methods

### Datasets

In this section, we describe the 2 public chest X-ray datasets used for this research: (I) HM Hospitals COVID-19 dataset “Covid data saves lives” and (II) RSNA Pneumonia Challenge dataset. Both are described in detail below.

#### HM hospitals COVID-19 dataset

**Fig. 2:**
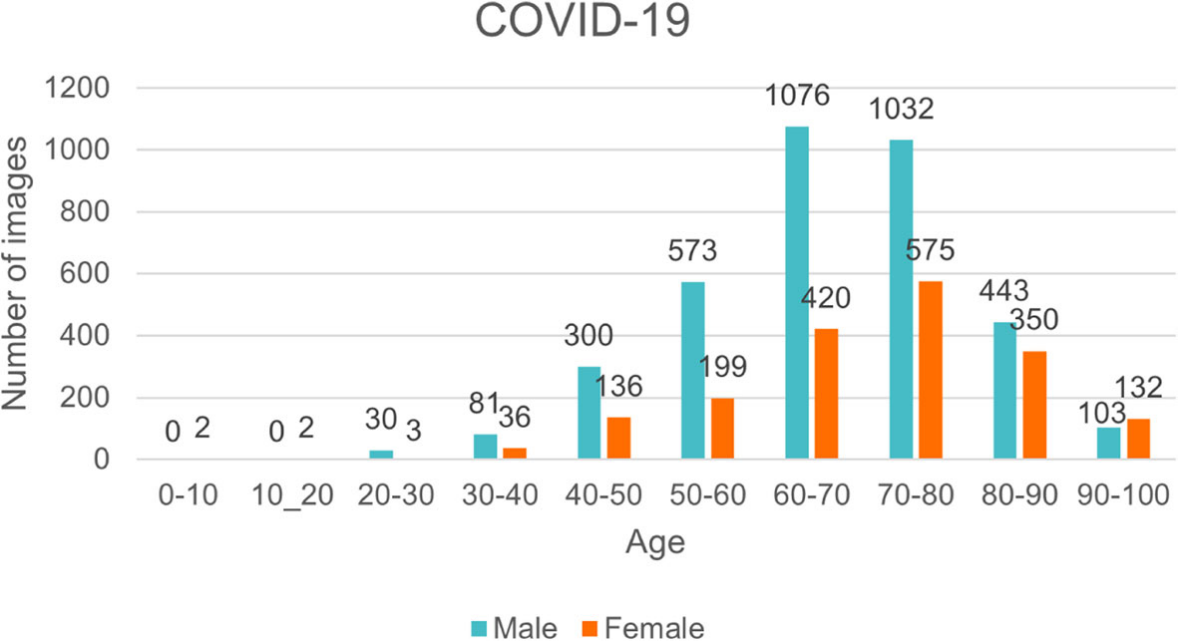
Age and sex distribution for chest X-ray images of the HM Hospitals COVID-19 dataset

HM Hospitals made available to the scientific community an anonymous dataset with all clinical information of patients treated in their hospitals by the COVID-19 virus [25]. This dataset is available upon request and must be approved by the HM Hospitals Research Ethics Committee. It consists of 2,310 patients with a diagnosis of “COVID-19 positive” or “COVID-19 pending” admitted to HM Hospitals. Chest X-rays are available for some of the patients, and these were taken during the time they were hospitalized. In this sense, we used 5,493 posteroanterior chest X-ray images from 1,832 different patients whose age and sex are distributed as indicated in Fig. 2 for our COVID-19 class.

#### RSNA Normal/Pneumonia dataset

**Fig. 3:**
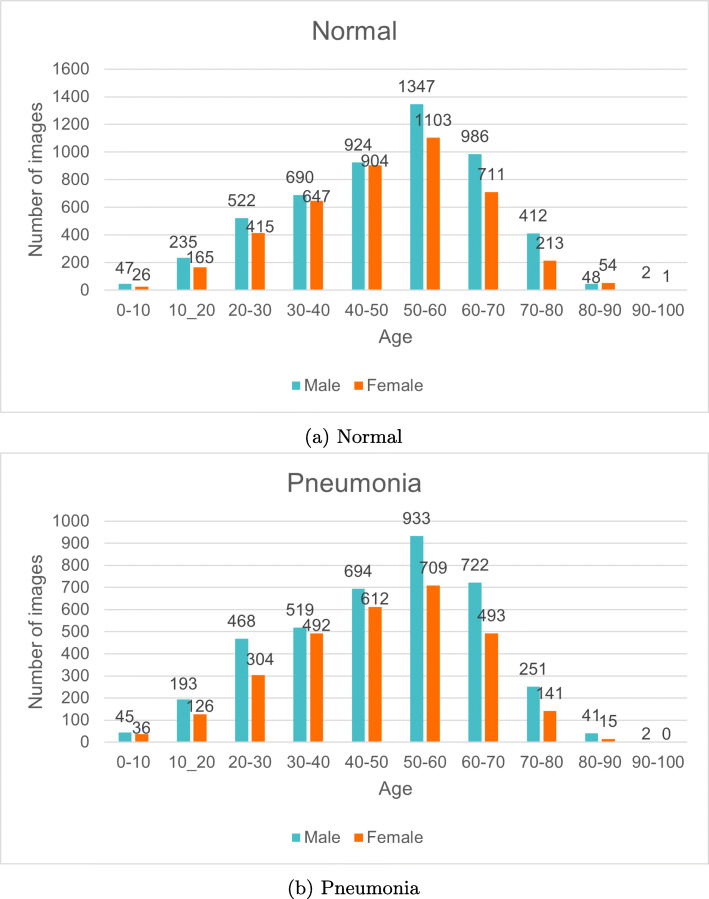
Age and sex distribution for chest X-ray images of the RSNA dataset

The RSNA Pneumonia Challenge dataset [26] is a subset of the ChestX-ray8 dataset [27] created for the Kaggle challenge on the MD.ai platform in collaboration with the Radiological Society of North America (RSNA). This dataset consists of 16,248 X-ray images, considering only the posteroanterior chest view, resulting in 9,452 images for normal cases and 6,796 images for patients diagnosed with pneumonia. In this dataset, we also have information about the age and sex of the patients. These characteristics are distributed in our subset as indicated in Fig. 3 for normal and pneumonia cases.

### Software and hardware resources

In this work, we used Python (version 3.6.6) for the implementation of the conducted studies and machine learning libraries PyTorch (version 0.4.1) and Scikit-learn (version 0.24.2) were used to train, validation and test the obtained models, as well as to get the metrics of their performances.

In addition, in order to facilitate the replication of our studies, we present in Table 1 the main specifications of the hardware used to perform the experiments.

**Table 1:** Specifications of the equipment used throughout the project to carry out the experiments

Name	Description
OS	DEBIAN GNU/Linux 10
Kernel	Linux 4.18.0-2-amd64
Architecture	x86-64
CPU	Intel(R) Xeon(R) CPU E5-2650 v4 @ 2.20GHz
Motherboard	Lenovo NeXtScale nx360 M5
RAM	16 GB de RAM GDDR5
HDD	IBM ServeRAID M5210 930 GB
GPU	NVIDIA Tesla P100
Driver Version	396.44
CUDA Version	9.2

### Architecture

In this work, we exploited the potential of the DenseNet-161 architecture [28]. This architecture is composed of dense blocks linked by transition layers, which in turn are formed by convolution and pooling layers. These dense blocks have layers with their own feature maps which consists of a batch normalisation operation, a ReLu operation and a 3 x 3 convolution with *k* filters, where *k* is the growth rate. Each of them receives the feature maps of all the previous layers, so that the collective knowledge of all the predecessor layers is preserved. In our case, this growth ratio *k* is 48, and the depth of the architecture *L* is 161. However, we modified its original structure to support the binary output defined in our computational approaches, as depicted in Table 2. This architecture provided satisfactory results in similar works aimed at classifying chest X-rays of patients with COVID-19 [17, 19, 20], which led us to choose it for this work.

**Table 2:** DenseNet-161 adapted structure

Layers	Output size	DenseNet-161
Convolution	112 x 112	Conv. 7 x 7, stride 2
Pooling	56 x 56	Max pool 3 x 3, stride 2
Dense block (1)	56 x 56	[1×1 *conv*. 3×3 *conv*.] x 6
Transition layer (1)	56 x 56	Conv. 1 x 1
	28 x 28	2 x 2 average pool, stride 2
Dense block (2)	28 x 28	[1×1 *conv*. 3×3 *conv*.] x 12
Transition layer (2)	28 x 28	Conv. 1 x 1
	14 x 14	2 x 2 average pool, stride 2
Dense block (3)	14 x 14	[1×1 *conv*. 3×3 *conv*.] x 36
Transition layer (3)	14 x 14	Conv. 1 x 1
	7 x 7	2 x 2 average pool, stride 2
Dense block (4)	7 x 7	[1×1 *conv*. 3×3 *conv*.] x 24
Classification layer	1 x 1	7 x 7 global average pool
		2D fully-connected, softmax

### Computational approaches for screening tasks

**Fig. 4:**
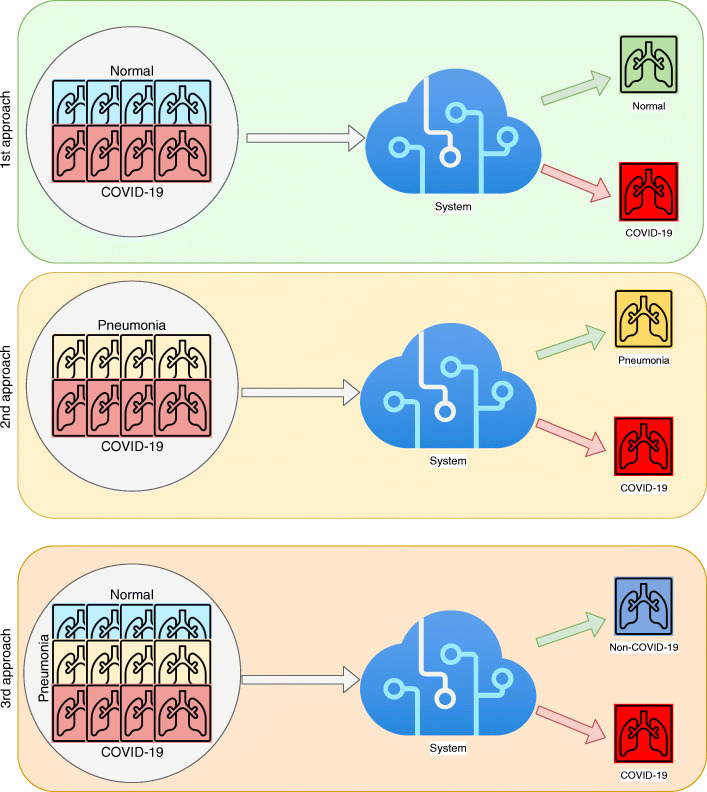
Schematic representation of computational approaches for COVID-19 screening using X-ray images

As illustrated in Fig. 4, we present 3 different approaches which classify X-ray images into 2 categories to differentiate COVID-19 patients from certain types of patients, as normal and pneumonia ones. Each of these approaches will be explained in more detail below, but in general these 3 different approaches cover a wide range of scenarios in which we can study in depth how gender and age factors affect the diagnosis of COVID-19 in deep learning systems. In this way, we will be able to draw more solid and contrasted conclusions, as most of the cases where a COVID-19 screening task is performed are taken into account and a bias could be more clearly detected.

#### 1^*st*^ approach: Normal vs. COVID-19

In this first scenario, we trained a model to obtain a consolidated approach to distinguish between normal cases (control patients without lung conditions but who may have other systemic pathologies) and COVID-19. We consider this scenario to be very useful as it is realistic and complex, as it is more difficult than distinguishing only between healthy patients and COVID-19. Moreover, this approach is present in the literature [29]. Both the fact that it is a situation that can occur in a clinical context and that it is a case that can be widely found in the state of the art make the casuistry present in this approach interesting when studying the influence of our target factors.

#### 2^*nd*^ approach: pneumonia vs. COVID-19

Given the similarities between COVID-19 and both viral and bacterial pneumonia, this second approach aims to differentiate between patients with COVID-19 and patients with pneumonia not caused by COVID-19. Thus, 2 different categories are predicted: pneumonia and COVID-19. Similar approaches have been studied in related works [12, 30]. Again, this is a complex situation that could be found in a real screening task and it is broadly studied in the state of art as well, so we find here a number of interesting cases where to explore the impact that sex and age could have.

#### 3^*rd*^ approach: non-COVID-19 vs. COVID-19

In this third approach, two categories are considered: one that has normal and pneumonia patients, named Non-COVID-19, and another one that has only COVID-19 patients. In this way, we can analyse the degree of separability between COVID-19 patients from all other cases. This kind of approach is common in related works [5, 7, 31]. Thus, this approach allows us, again, to investigate how our target factors could affect a wide number of real and complex cases taken into account here.

### Training details

The final dataset for each experiment where we will study the sex and age factors was divided into mutually exclusive subsets, being (60%, 20%, and 20%) for training, validation, and testing, respectively. Regarding the training, we started from the DenseNet-161 model pre-trained with the ImageNet [32] dataset, making use of the transfer learning strategy, but modifying the output layer to adapt it to our specific classification problem. In this way, the training process will be more efficient due to the faster convergence of the training and validation curves. It also reduces the number of labeled images necessary for the process to be adequate [24]. On the other hand, a cross-entropy loss function is performed on the output class and the ground truth for the target X-ray image. The optimization during the training is carried out by Stochastic Gradient Descent (SGD) [33] with a learning rate constant of 0.01, a mini-batch size of 4, and a first-order momentum of 0.9, all of them obtained by exhaustive experimentation. This optimiser has proven to be very efficient, despite its simplicity, for the discriminative learning of classifiers under convex loss functions, defined as follows, where *Y* represents the ground truth values and $\hat {Y}$ represents the estimated values for each identified category:

1 $$ L = -Y \cdot log(\hat{Y})  $$

A complete training epoch includes a run through all the samples of the training set. Each training process had 200 epochs, since a larger number of epochs would not produce of epochs did not produce significant improvements neither in the loss function nor in the accuracy metrics. In addition, to ensure the generalization capability of the approaches presented, each experiment was repeated 5 times independently of each other with random sample selection, so it was necessary to calculate the means of these repetitions to evaluate the overall global performance. To compensate for the lack of available X-ray images and thus avoid problems of overfitting and to increase the generalization capacity, data augmentation was performed to obtain more robust and stable models. Thus, scaling and horizontal rotation operations were performed, which are appropriated given the symmetrical nature of the chest X-ray image, so the variability of the data used was increased. We consider this configuration to be suitable enough for our sex and age study, as it has provided satisfactory results in similar works [17, 19, 20].

### Evaluation

The performance of the presented computational approaches was evaluated by comparing the predictions provided by the models with the ground truth labels annotated in the X-ray image datasets. Then, the values of True Positives (TP), True Negatives (TN), False Positives (FP) and False Negatives (FN) were considered to calculate different metrics that are commonly used in the literature [17, 19, 20] to assess the stability of computational methods for medical imaging problems. Following the reference of these similar works, we also decided to use these metrics for our analysis of the sex and age factors. Thus, Precision, Recall, F1-score, and Accuracy were calculated for each approach as follows.

2 $$ Precision = \frac{TP}{TP + FP}  $$

3 $$ Recall = \frac{TP}{TP + FN}  $$

4 $$ F1-score = 2 * \frac{Precision * Recall}{Precision + Recall}  $$

5 $$ Accuracy = \frac{TP + TN}{TP + TN + FP + FN}  $$

## Results

In this section, we present the experimental results of the proposed computational approaches for the classification of COVID-19 in chest X-ray images, covering a wide range of cases that will allow us to draw more contrasted and solid conclusions regarding the studied factors of sex and age. In particular, we perform two different and complementary studies on the COVID-19 dataset. The first one analyses the influence of the sex factor for each of the 3 approaches: (I) Normal VS COVID-19, (II) Pneumonia VS COVID-19 and (III) Non-COVID-19 VS COVID-19. The second one performs a similar analysis, but in this case considering patients by age ranges. Both studies are described below.

### Sex-related imbalance analysis

**Fig. 5:**
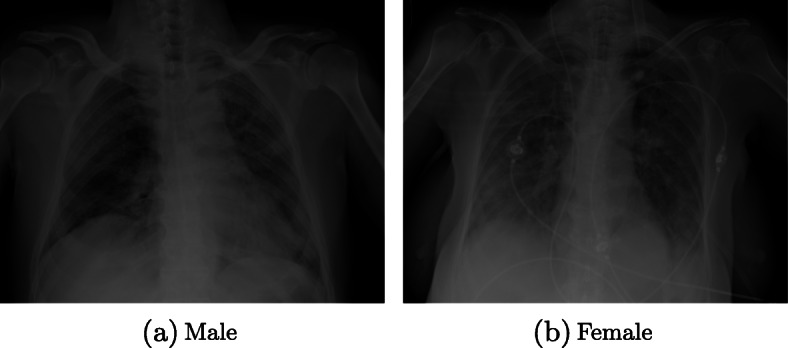
Example of two representative chest X-ray images of male and female patients diagnosed with COVID-19

One of the main characteristics of a patient that can influence a diagnostic system is sex [22, 23]. Especially in chest x-rays, we might think that differences in size, in addition to other typical sex characteristics such as the presence of breasts, could imply taking the images in different postures or certain abnormalities in the samples that could be mistaken for signs of a pathology, in this case this being COVID-19 [34]. In Fig. 5, we exemplify these differences with 2 patients of different sexes who have COVID-19. Considering how important is to identify a bias related to the sex of the patient, we designed the following study in order to test whether this characteristic influences diagnosing COVID-19.

In this first analysis, we explored intermediate imbalance scenarios in which female and male patients diagnosed with COVID-19 were analysed in different proportions with 10% intervals, ranging from 0% male patients and 100% female patients to 100% male patients and 0% female patients. Thus, we conducted a comprehensive analysis with 11 different configurations for each computational approach. For each imbalance case, we get a model that is then tested using the remaining images not used during training. Afterwards, we compare the results obtained for each scenario with our baseline (50% female and 50% male). Regarding the amount of images considered for each approach, we used 700 COVID-19 images from 700 different patients. Although the dataset considered in this study consists of 5,493 COVID-19 images, it includes several COVID-19 images obtained from the same patient over time. Furthermore, in terms of gender, the dataset is composed of 1,132 male and 730 female patients. Finally, we have discarded 30 female patients because they did not have a chest X-ray image but another type of medical image, such as a lung CT scan. Therefore, in order to perform a more honest and unbiased analysis, we only have 700 patients in sex-related imbalance analysis. To maintain balance between this COVID-19 class and the other classes, 700 X-ray images were randomly selected and divided according to the sex of the patient, as indicated in Table 3. Therefore, each of the 11 experiments was performed using 1400 chest X-ray images.

**Table 3:** Distribution of randomly selected X-ray images for each computational approach in the sex-related imbalance analysis

Approach	Normal	Pneumonia
Normal VS COVID-19	350 M + 350 F	0
Pneumonia VS COVID-19	0	350 M + 350 F
Non-COVID-19 VS COVID-19	175 M + 175 F	175 M + 175 F

#### Analysis of the 1^*st*^ approach: Normal vs. COVID-19

In Table 4 we present a comparative analysis of the performance at the test stage using precision, recall, and F1-score measures, where we highlight our baseline as we are going to use it to compare our metrics. As for the mean accuracy obtained at each scenario, our values ranged from 0.9757 ± 0.0105 at the 40%M 60%F case, to 0.9835 ± 0.0105 at the 90%M 10%F case. The standard deviation of these metrics was always below 2.1%, being the highest at the 60%M 40%F case, and the lowest at 30%M 70%F with 0.58%. In general, it can be observed that the differences between the metrics are small when compared to our baseline and their values are maintained regardless of the studied scenario.

**Table 4:**
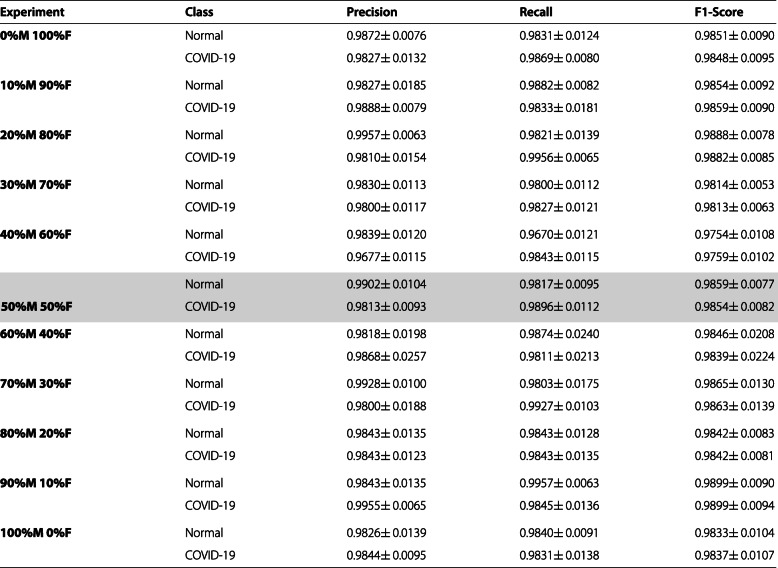
Mean ± standard deviation of the results obtained in the test stage for the classification of chest X-ray images between Normal VS COVID-19 after 5 independent repetitions. The baseline is highlighted in grey

#### Analysis of the 2^*nd*^ approach: pneumonia vs. COVID-19

The second group of experiments deals with the analysis of sex-related imbalance in the second approach. In this line, Table 5 show a comparative analysis of the performance at the test stage using precision, recall, and F1-score measures. Here, we highlight our baseline as we are going to use it to compare our metrics. As we can see, the results show a similar tendency to the previous set of experiments of the first approach, with values for the mean accuracy ranged from 0.9721 ± 0.0187 at the 0%M 100%F case, to 0.9892 ± 0.0091 at the 100%M 0%F case. The standard deviation of these metrics was always below 1.8%, being the highest at the 0%M 100%F case, and the lowest at 10%M 90%F with 0.86%.

**Table 5:**
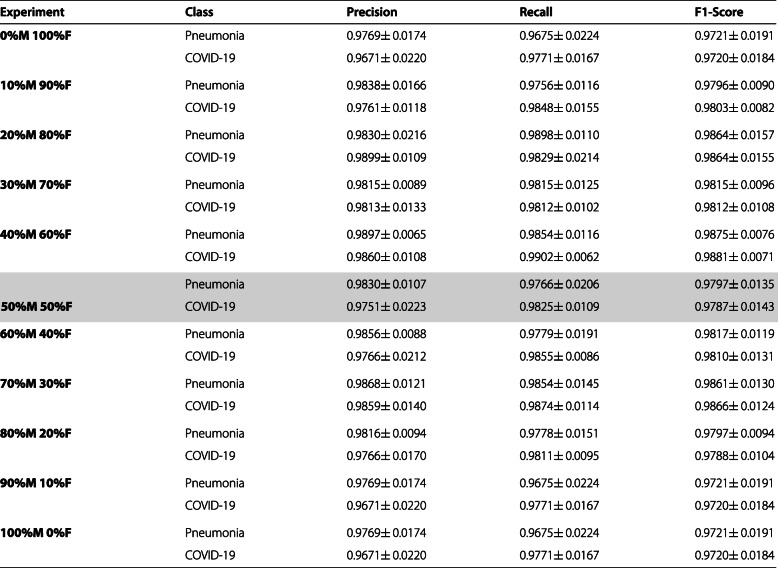
Mean ± standard deviation of the results obtained in the test stage for the classification of chest X-ray images between Pneumonia VS COVID-19 after 5 independent repetitions. The baseline is highlighted in grey

#### Analysis of the 3^*rd*^ approach: non-COVID-19 vs. COVID-19

In this third set of experiments, we analyzed the behavior of the sex factor imbalance in the data on separability between the Non-COVID-19 vs. COVID-19 classes. Table 6 shows the results of the test stage in terms of precision, recall and F1-Score for each class, after performing the proposed experiments, and we highlighted our baseline as we are going to use it to compare our metrics. As we can see, these results reflect that all models are able to accurately separate samples from both classes. As for the mean accuracy obtained at each scenario, our values ranged from 0.9700 ± 0.0117 at the 40%M 60%F case, to 0.9857 ± 0.0035 at the 100%M 0%F case. The standard deviation of these metrics was always below 1.3%, being the highest at the 60%M 40%F case, and the lowest at 100%M 0%F with 0.35%.

**Table 6:**
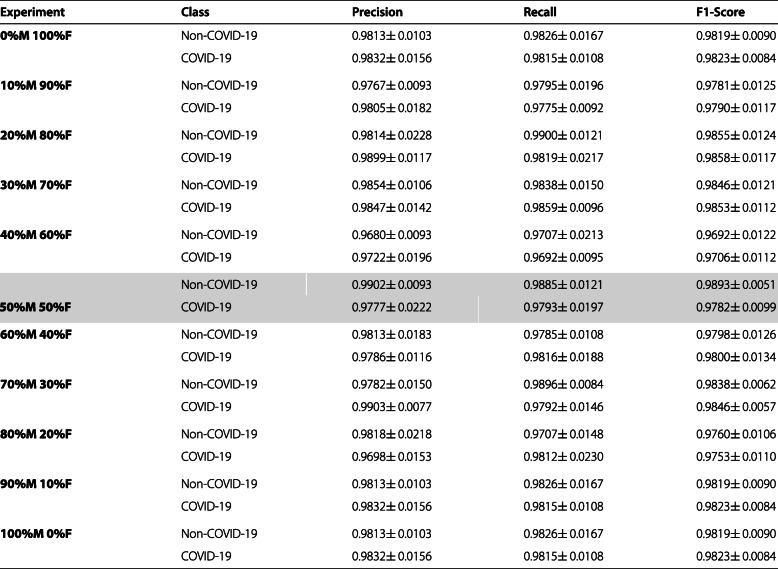
Mean ± standard deviation of the results obtained in the test stage for the classification of chest X-ray images between Non-COVID-19 VS COVID-19 after 5 independent repetitions. The baseline is highlighted in grey

### Age-related imbalance analysis

**Fig. 6:**
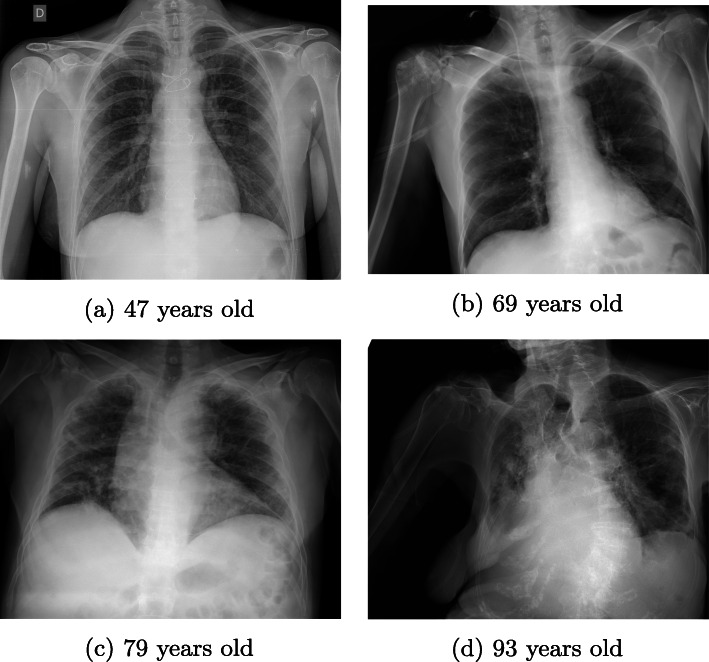
Example of four representative chest X-ray images of patients of different ages diagnosed with COVID-19

Age-related deterioration of both the skeleton and the musculature of the body is visible on chest X-rays, which may affect the diagnosis obtained from them [22, 35]. In addition, older COVID-19 patients often require more medical equipment that appears on chest X-ray images, such as intravenous lines, ventilators, pacemakers, and so on, which may again affect the diagnosis obtained from the X-rays [34]. To illustrate these characteristics associated with different ages, Fig. 6 shows representative examples of different COVID-19 patients ranging in age from 47 to 93 years old. These differences raise the need for a detailed study of how the patient age affects the diagnosis of COVID-19. Therefore, we describe below the analysis we have carried out for this purpose.

For the age-related imbalance study, we defined 6 different age ranges: 0-40, 40-50, 50-60, 60-70, 70-80, ≥ 80. For each range, we used only images from patients in that age spectrum for training and then tested it with the remaining images. We analysed the differences between the age group used for training, which acts as our baseline, and all other ages. Regarding the exact number of samples used for each class in our 3 computational approaches, we present our distribution in Table 7. Using this amount of images of each class, we sought to emphasise the older age groups, who suffer more from the disease and have to go through a more critical diagnostic process, but also adapting to the number of samples we had available from the studied Normal, Pneumonia and COVID-19 classes of interest.

**Table 7:** Number of samples of each class considered per approach

Age	Normal VS COVID-19	Pneumonia VS COVID-19	Non-COVID-19 VS COVID-19
<40	154 vs 154	154 vs 154	(77+77) vs 154
40-50	436 vs 436	436 vs 436	(218+218) vs 436
50-60	772 vs 772	772 vs 772	(386 + 386) vs 772
60-70	1,496 vs 1,496	1,215 vs 1,215	(748+748) vs 1,496
70-80	625 vs 625	392 vs 392	(392+392) vs 784
≥ 80	105 vs 105	58 vs 58	(58+58) vs 116

In the following sections, we will show the results of our six baselines (one per age range) for each approach. However, the details of how these baselines responded to the different age groups will be discussed in the Discussion section in order to simplify this section and facilitate understanding.

#### Analysis of the 1^*st*^ approach: Normal vs. COVID-19

For this first approach, we present in Table 8 precision, recall and F1-score means and their standard deviation obtained at test for each experiment training with only one age group. These results for our six baselines were satisfactory and mainly stable, as the metrics were over 90% in most cases and standard deviation was under 8%. Regarding the mean accuracy obtained for each one of these baselines, we obtained the following values: 0.9587 ± 0.0298, 0.9748 ± 0.0012, 0.9877 ± 0.0001, 0.9876 ± 0.0001, 0.9808 ± 0.0004 and 0.9429 ± 0.0086, ordering them from the youngest to the oldest age group. In general, this indicates that our baselines are acceptable and stable, since the accuracy was above 94% and the standard deviation kept under 8.6%.

**Table 8:** Mean ± standard deviation of the results obtained in the test stage for the classification of chest X-ray images between Normal VS COVID-19 after 5 independent repetitions

Exp.	Class	Precision	Recall	F1-Score
<40	Normal	0.9453 ± 0.0514	0.9749 ± 0.0349	0.9588 ± 0.0277
	COVID-19	0.9742 ± 0.0355	0.9438 ± 0.0588	0.9573 ± 0.0276
40-50	Normal	0.9673 ± 0.0288	0.9814 ± 0.0141	0.9742 ± 0.0193
	COVID-19	0.9816 ± 0.0125	0.9693 ± 0.0250	0.9753 ± 0.0156
50-60	Normal	0.9911 ± 0.0057	0.9846 ± 0.0080	0.9878 ± 0.0053
	COVID-19	0.9844 ± 0.0066	0.9907 ± 0.0058	0.9875 ± 0.0043
60-70	Normal	0.9925 ± 0.0055	0.9826 ± 0.0062	0.9875 ± 0.0053
	COVID-19	0.9828 ± 0.0064	0.9926 ± 0.0055	0.9877 ± 0.0054
70-80	Normal	0.9780 ± 0.0171	0.9846 ± 0.0086	0.9812 ± 0.0102
	COVID-19	0.9832 ± 0.0111	0.9774 ± 0.0173	0.9802 ± 0.0115
≥ 80	Normal	0.9373 ± 0.0691	0.8912 ± 0.0849	0.9125 ± 0.0690
	COVID-19	0.8859 ± 0.0805	0.9255 ± 0.0800	0.9043 ± 0.0734

#### Analysis of the 2^*nd*^ approach: pneumonia vs. COVID-19

For our second set of experiments, we summarized in Table 9 the metrics and their standard deviation obtained for our baseline models at the test stage for each experiment training with only one age group. Again, these models had acceptable results, as they were above 90% in nearly all cases and its standard deviations were below 10%. As for the mean accuracy obtained for each one of these baselines, we obtained these values for every baseline ordered by age: 0.9396 ± 0.0027, 0.9760 ± 0.0004, 0.9800 ± 0.0005, 0.9919 ± 0.0001, 0.9772 ± 0.0004 and 0.9083 ± 0.043. Overall, these metrics are satisfactory and steady, being above 90% and with a standard deviation under 4.3%.

**Table 9:** Mean ± standard deviation of the results obtained in the test stage for the classification of chest X-ray images between Pneumonia VS COVID-19 after 5 independent repetitions

Exp.	Class	Precision	Recall	F1-Score
<40	Pneumonia	0.9603 ± 0.0440	0.9292 ± 0.0241	0.9438 ± 0.0188
	COVID-19	0.9199 ± 0.0303	0.9498 ± 0.0625	0.9336 ± 0.0356
40-50	Pneumonia	0.9811 ± 0.0177	0.9717 ± 0.0161	0.9762 ± 0.0105
	COVID-19	0.9689 ± 0.0219	0.9821 ± 0.0172	0.9752 ± 0.0122
50-60	Pneumonia	0.9802 ± 0.0118	0.9815 ± 0.0116	0.9808 ± 0.0112
	COVID-19	0.9796 ± 0.0126	0.9784 ± 0.0129	0.9790 ± 0.0122
60-70	Pneumonia	0.9942 ± 0.0046	0.9895 ± 0.0036	0.9918 ± 0.0036
	COVID-19	0.9893 ± 0.0040	0.9944 ± 0.0045	0.9919 ± 0.0035
70-80	Pneumonia	0.9869 ± 0.0132	0.9678 ± 0.0101	0.9772 ± 0.0097
	COVID-19	0.9668 ± 0.0123	0.9874 ± 0.0122	0.9770 ± 0.0097
≥ 80	Pneumonia	0.8850 ± 0.1117	0.9000 ± 0.1732	0.8893 ± 0.1380
	COVID-19	0.9346 ± 0.1084	0.9095 ± 0.0831	0.9195 ± 0.0840

#### Analysis of the 3^*rd*^ approach: non-COVID-19 vs. COVID-19

Finally for this third approach, we show in Table 10 precision, recall and F1-score means and their standard deviation obtained at test for each experiment training with only one age group. Following the trend that we have already seen in the two previous approaches, our baseline models had adequate metrics, as they were above 90% in all scenarios and the corresponding standard deviation was below 6%. The results obtained for the mean accuracy from the youngest to the oldest baseline were the following: 0.9683 ± 0.0020, 0.9760 ± 0.0002, 0.9819 ± 0.0002, 0.9913 ±0.8×10^−5^, 0.9898 ±0.8×10^−4^ and 0.9234 ± 0.0077. As we can see, all baselines remained above 96% and their standard deviation was under 7.7%, which make these metrics satisfactory and mainly stable.

**Table 10:** Mean ± standard deviation of the results obtained in the test stage for the classification of chest X-ray images between Non-COVID-19 VS COVID-19 after 5 independent repetitions

Exp.	Class	Precision	Recall	F1-Score
<40	Non-COVID-19	0.9754 ± 0.0141	0.9625 ± 0.0344	0.9688 ± 0.0231
	COVID-19	0.9617 ± 0.0352	0.9725 ± 0.0157	0.9669 ± 0.0226
40-50	Non-COVID-19	0.9707 ± 0.0155	0.9821 ± 0.0154	0.9762 ± 0.0059
	COVID-19	0.9812 ± 0.0189	0.9701 ± 0.0184	0.9754 ± 0.0093
50-60	Non-COVID-19	0.9849 ± 0.0096	0.9802 ± 0.0105	0.9825 ± 0.0078
	COVID-19	0.9785 ± 0.0123	0.9840 ± 0.0096	0.9812 ± 0.0084
60-70	Non-COVID-19	0.9925 ± 0.0043	0.9898 ± 0.0041	0.9911 ± 0.0016
	COVID-19	0.9901 ± 0.0039	0.9927 ± 0.0043	0.9914 ± 0.0011
70-80	Non-COVID-19	0.9950 ± 0.0052	0.9850 ± 0.0069	0.9900 ± 0.0046
	COVID-19	0.9846 ± 0.0072	0.9947 ± 0.0053	0.9896 ± 0.0047
≥ 80	Non-COVID-19	0.9429 ± 0.0547	0.9107 ± 0.0633	0.9250 ± 0.0426
	COVID-19	0.9068 ± 0.0573	0.9380 ± 0.0695	0.9206 ± 0.0480

## Discussion

**Fig. 7:**
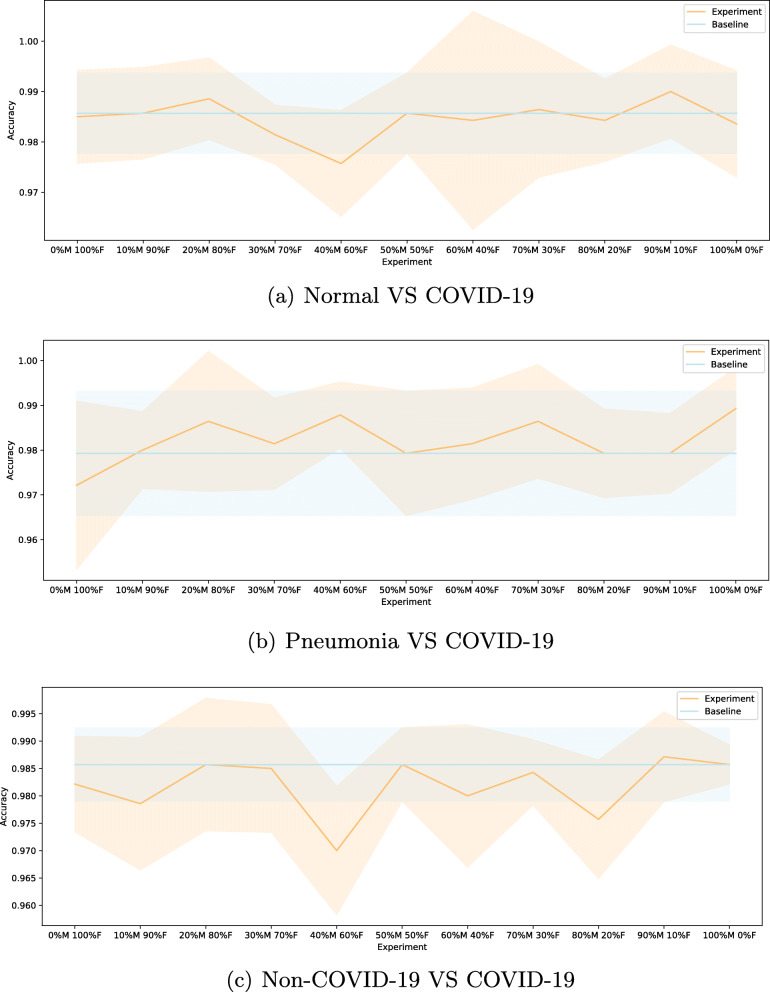
Mean ± standard deviation test accuracy obtained for every studied scenario in every approach

Regarding the sex-related imbalance analysis, the precision, recall and F1-score measures shown in Results section were in every experiment in all the approaches above 96%, which is a satisfactory result. As for accuracy, we summarized the obtained measures for every experiment for each approach in Fig. 7. We can see here how there are no extreme peaks in either the accuracy or its standard deviation in none of the approaches, and differences between experiments and approaches are around 5%. Although the Normal VS COVID-19 approach has a bigger standard deviation peak at the 60% male and 40% female experiment, all values remain closer and similar to our baseline. The same occurs for the Pneumonia VS COVID-19 approach, as accuracy continues to be stable and alike our baseline. In the Non-COVID-19 VS COVID-19 approach we have a slightly different scenario, since most of the obtained values are under our baseline, especially in experiments 40% male and 60% female, and 80% male and 20% female. Despite these differences, we can observe how accuracy remains stable and similar to other approaches. All these satisfactory results, together with the stability observed in all the scenarios considered in each of our approaches, indicate that this factor has not clearly affected the diagnosis offered by our system. If it had, we would have seen graphs with more evident differences between each of the different sex ratios with which we experimented. Thereby, no influence caused by the sex factor was observed. Although male and female patients may have differentiating features that allow us to identify their sex on chest x-rays, such as breasts, differences in shape and size, etc., these typically sex-associated features do not influence their COVID-19 diagnosis and do not favour one sex over the other, as they do not interfere with the lung assessment. For example, differences in shape and size do not difficult the finding of suspicious densities in the lung itself, and those densities related to the mammary glands are easily discarded, as they are present in most female patients and do not usually obscure COVID-19 related findings.

**Fig. 8:**
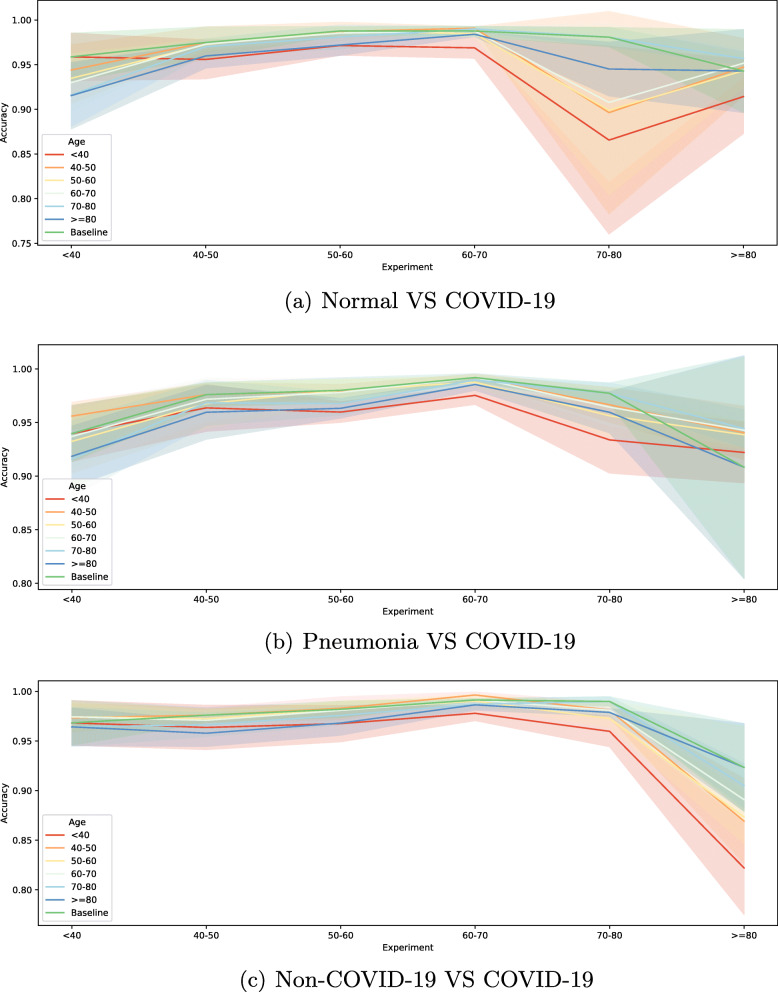
Mean ± standard deviation test accuracy obtained for every studied age range in every approach

Regarding the age-related imbalance analysis, the precision, recall and F1-score measures shown in Results section were in every experiment in all approaches above 96%, which is a satisfactory result. As for accuracy, we summarized the obtained results for each approach in Fig. 8, taking as a reference the baselines metrics shown in the Results section. In this accuracy comparative across all six age ranges it is presented how its standard deviation increases as baseline patients get older than 70. The worst instability peaks are in the 70-80 range in the Normal VS COVID-19 approach and the ≥ 80 range in the Pneumonia VS COVID-19 approach, but these increases only represent a worsening of 10%. This behaviour is not as clearly observed for the Non-COVID-19 VS COVID-19 approach, since its standard deviations rises at the ≥ 80 range, but not as noticeably as in other approaches. In relation to the accuracy metric itself, it is observed how the closer to the baseline age the tested age range gets, the better accuracies are obtained. However, these differences are not of great magnitude. In general, the third approach seems like the best and most stable of the three ones considered, since its accuracy is consistently good enough at every age range, and its standard deviation has a smaller peak at the older ages. Nevertheless, both the worsening in the obtained accuracy and the its instability are not of great magnitude in any approach. Thus, we can clearly observe in these graphs the clear tendency of the diagnosis offered to be influenced by age, regardless of the age group studied or the used computational approach. Moreover, it is noteworthy that this worsening is more or less present in all the cases studied, but is more pronounced in the older age groups, which is consistent given that the most critical cases of COVID-19 are more frequent in this group, resulting in a greater variability of pathological affectations in the lungs. For example, older patients are usually easily recognized by the wide range of different damaged ribcages they might present, being these caused by diseases or by the passing of time. In this situation, these patients are typically weaker in the face of such an aggressive disease as COVID-19, so different types of medical equipment, such as pathways or thoracostomy tubes, among other cardiac and pulmonary devices, are more present in these X-rays. All of these elements can appear on these images, obscuring lung densities typical of COVID-19 or leading our systems to recognise these patients more by the irregularity of their X-rays than by the signs of disease they may manifest, both affecting their COVID-19 diagnosis. However, these characteristics do not appear as frequently in the chest X-rays of younger patients, who typically have images where abnormalities are more easily observed and their association to COVID-19 is more straightforward, because they do not have other pathologies that may cause the presence of irregularities in their images. Hence, these reasons could justify the presence of this bias. In this work, we have performed a comprehensive analysis of sex and age factors in the chest Xray images. Accordingly, we have generated 615 ROC curves from the experiments (see supplementary material available at 10.1186/s12874-022-01578-w).

## Conclusions

In this work, we have proposed the first study to analyze whether imbalance in chest X-ray datasets produces biased deep learning approaches for COVID-19 screening with respect to the studied sex and age factors. For this purpose, 3 computational approaches using deep learning strategies that allowed us to carry out these studies of these factors in a detailed and comprehensive manner are presented and evaluated. To demonstrate the capabilities of our proposal, we perform several experiments on different public image datasets, including Normal, Pneumonia and COVID-19 cases. The presented results evidenced that the proposed methodology and tested approaches allow a robust and reliable analysis to support the clinical decision-making process in this pandemic scenario. Given the effort made to consider as many cases as possible and to make these studies as comprehensive as possible, we believe that the conclusions presented below are robust and reliable.

Regarding the sex-related imbalance analysis, we observed that this characteristic did not significantly affect the performance of our system. Whatever the sex ratio, the system performed well and provided satisfactory and stable results in all analyzed approaches. Since we performed a thorough study where we examined many different scenarios and explored different sex proportions, we can conclude that our system was not biased by this characteristic. Therefore, any difference observed between male and female patients from our dataset was not big enough to influence the system. On the other hand, regarding the age-related imbalance analysis, we observed that this characteristic did affect the performance of our system. It was clearly seen in every approach how the age used for training biased the system making it perform better for those with closer ages to the training phase one. Although obtained accuracy was good enough in every scenario as it was above 90% for most of the cases, age bias was consistent across all approaches. Again, since this analysis was conducted in a comprehensive manner, we can reliably conclude that the system was affected by the age of the patient. This could be caused by many reasons. For example, older patients have more irregular chest X-rays than younger people, since they can manifest different bone or cardiac pathologies. These differences might explain separability between the age ranges studied and their different results. Despite the fact a clear cause for this behaviour was not found, it is not necessary to emphasize how much it is needed to review the datasets being used for COVID-19 screening and identify possible bias related to the patient’s age in them, since it was checked by our experiments that this factor’s imbalance might affect the performance of the developed system.

As future work, it would be interesting to extend our study with patients diagnosed with other pulmonary disorders, such as emphysema, bronchitis and tuberculosis, among others. On the one hand, common pathologies affecting the lungs could represent a more challenging scenario of interest. On the other hand, expanding the dataset is of great interest to validate more completely the proposed methodologies. Other interesting future work would be to extend this analysis to other types of medical imaging modalities and correlate the results in a multimodal context to identify more precisely the influence of sex and age factors in COVID-19 screening systems. From a more technical point of view, in this work, we choose the input image size that is commonly used in the state of the art in similar problems. However, analyzing the relevance of this factor would ensure that important details are not being overlooked by reducing the image so much. In this sense, a more complete study could be done, testing with different input sizes. In addition, to facilitate the detection of biases of this type in related works, it would be interesting to implement a graphical user interface in order to make it easier for other users to test our methodology with different datasets.

## Supplementary Information

**Additional file 1** Supplementary material.

## Data Availability

The data that support the findings of this study are available from Radiological Society of North America (RSNA) and the HM Hospitales group. The RSNA dataset is publicly available in the Kaggle platform (https://www.kaggle.com/c/rsna-pneumonia-detection-challenge). In the case of the HM Hospitales dataset, although publicly available, restrictions apply to the availability of these data, which were used under licence for the present study. The source code developed during this study is available from the corresponding author on reasonable request.

## Abbreviations

WHO: World Health Organization
SARS-CoV-2: Severe Acute Respiratory Syndrome Coronavirus 2
CNN: Convolutional Neural Network
CAAD: Confidence Aware Anomaly Detection
TP: True Positive
TN: True Negative
FP: False Positive
FN: False Negative
