# Supplementary material for: Does imbalance in chest X-ray datasets produce biased deep learning approaches for COVID-19 screening?
Source: BMC Med Res Methodol. 2022 Apr 28;22:125. doi: 10.1186/s12874-022-01578-w (PMC9046709; doi:10.1186/s12874-022-01578-w)
Supplement: Supplementary file 1 — Additional file 1 Supplementary material. [file 12874_2022_1578_MOESM1_ESM.pdf]

# ROC graphs for sex and age imbalance analysis

## 1 Sex-imbalance analysis

### 1.1 Approach 1: Normal VS COVID-19

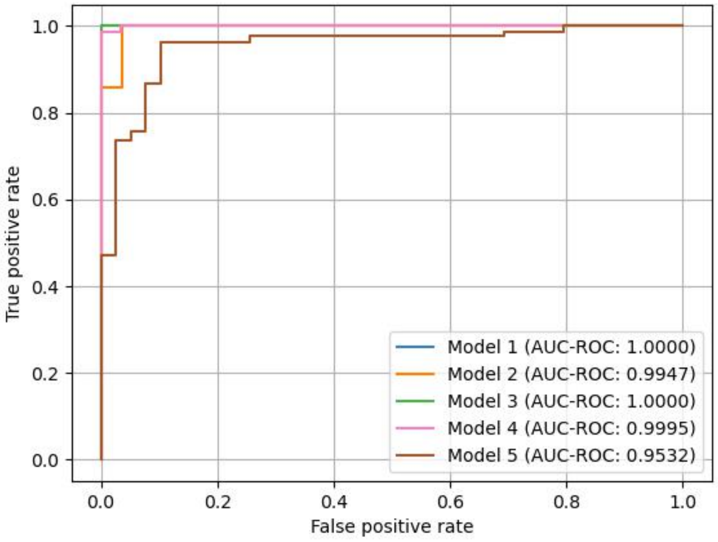


Figure S1: ROC for sex experiment 0%M 100%F (Normal VS COVID-19).

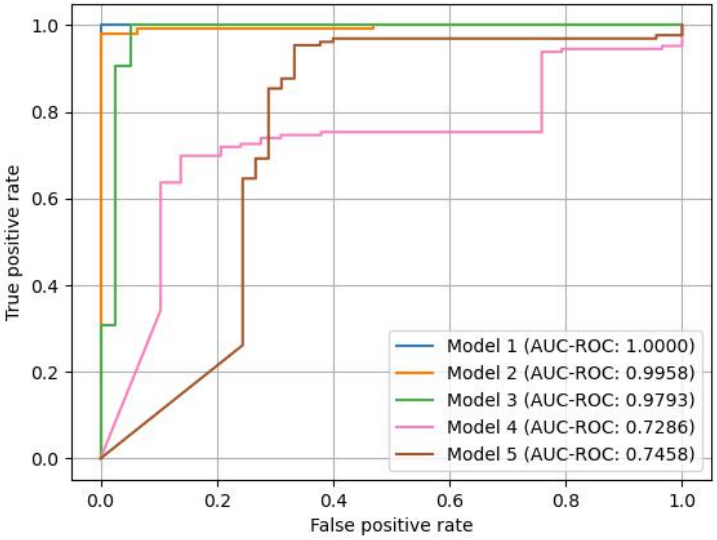


Figure S2: ROC for sex experiment 10%M 90%F (Normal VS COVID-19).

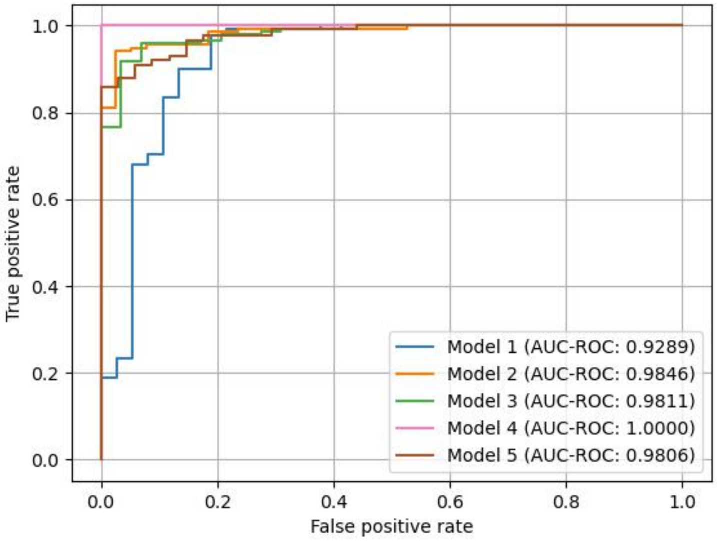


Figure S3: ROC for sex experiment 20%M 80%F (Normal VS COVID-19).

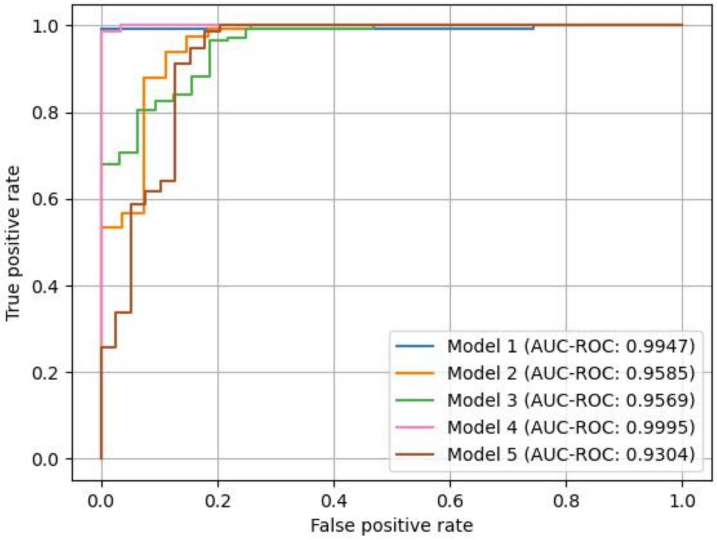


Figure S4: ROC for sex experiment 30%M 70%F (Normal VS COVID-19).

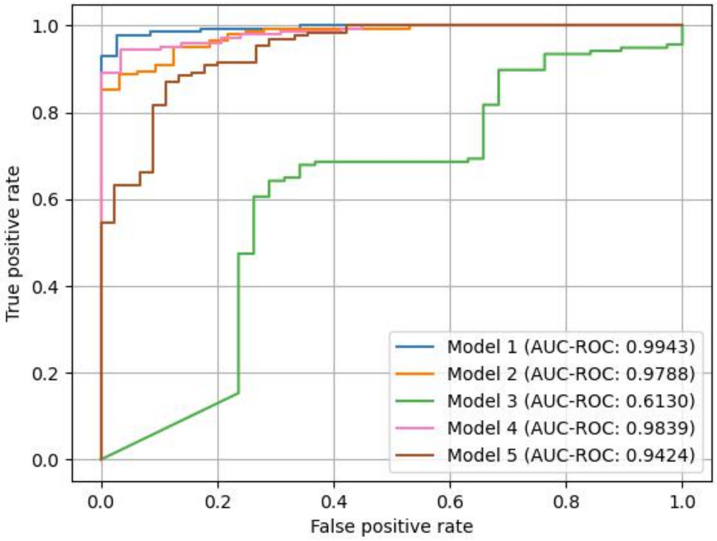


Figure S5: ROC for sex experiment 40%M 60%F (Normal VS COVID-19).

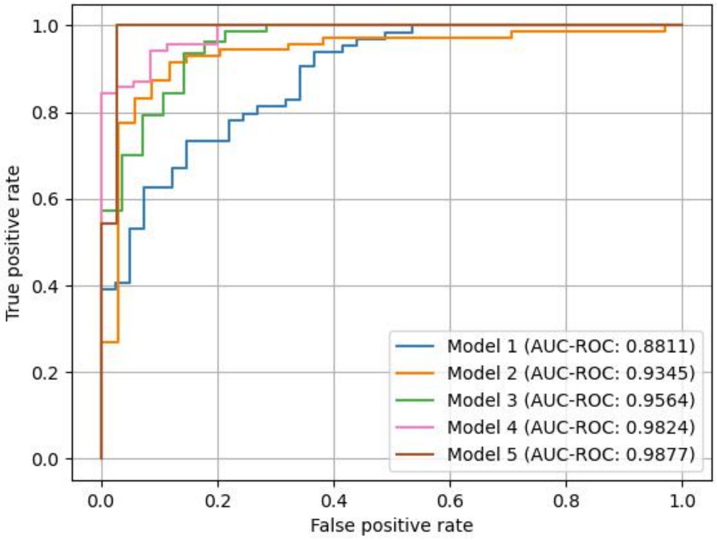


Figure S6: ROC for sex experiment 50%M 50%F (Normal VS COVID-19).

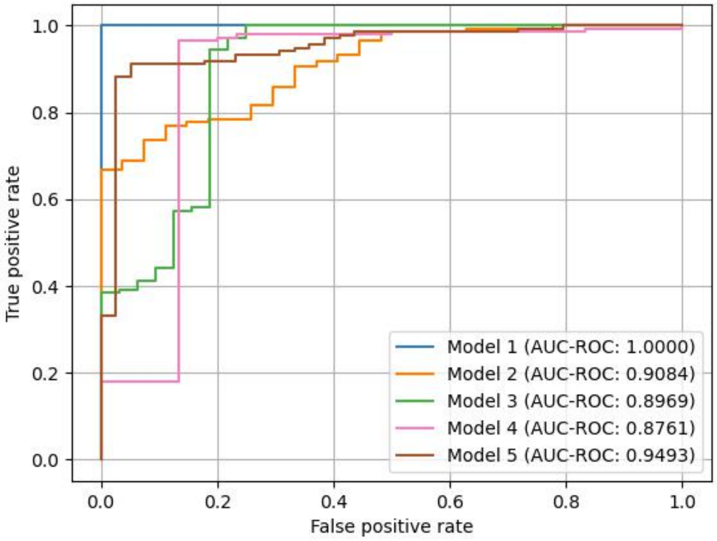


Figure S7: ROC for sex experiment 60%M 40%F (Normal VS COVID-19).

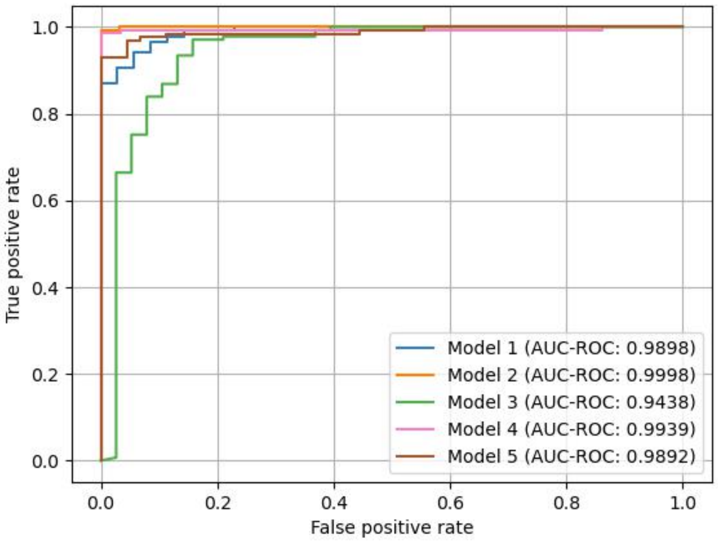


Figure S8: ROC for sex experiment 70%M 30%F (Normal VS COVID-19).

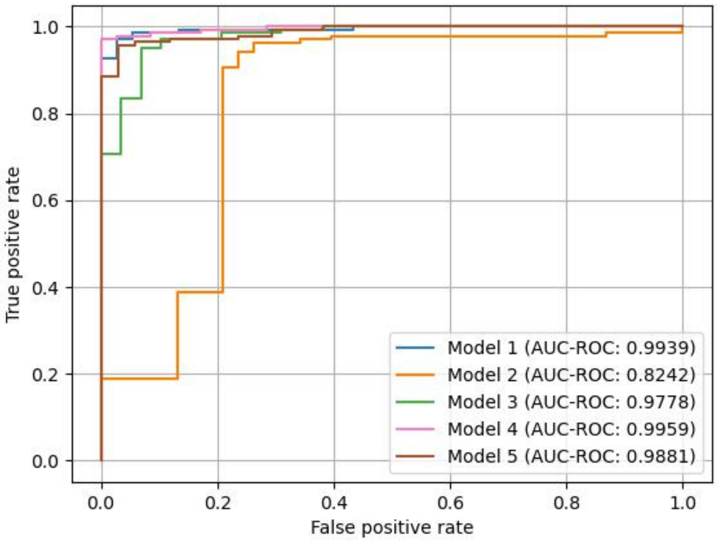


Figure S9: ROC for sex experiment 80%M 20%F (Normal VS COVID-19).

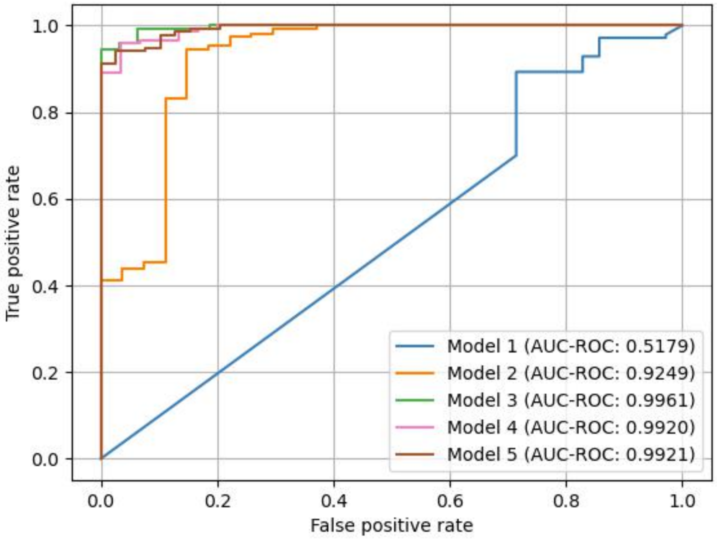


Figure S10: ROC for sex experiment 90%M 10%F (Normal VS COVID-19).

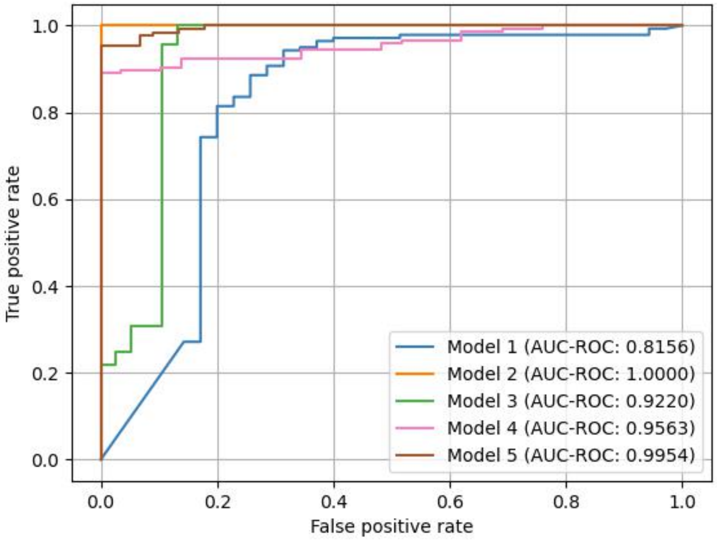


Figure S11: ROC for sex experiment 100%M 0%F (Normal VS COVID-19).

## 1.2 Approach 2: Pneumonia VS COVID-19

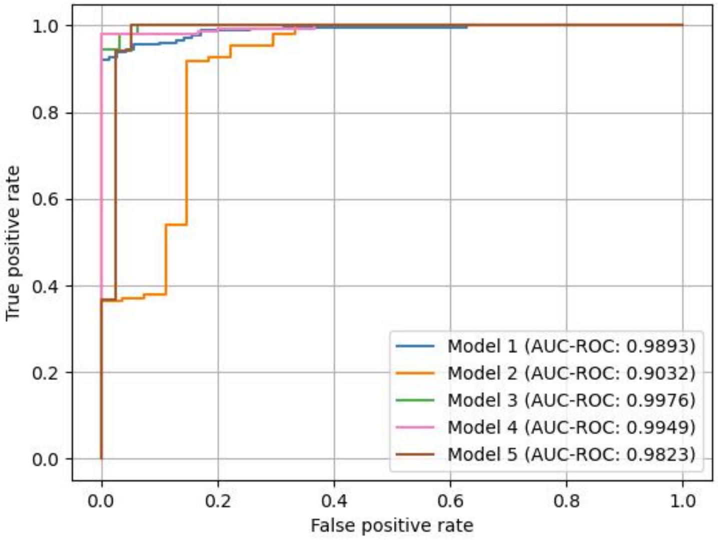


Figure S12: ROC for sex experiment 0%M 100%F (Pneumonia VS COVID-19).

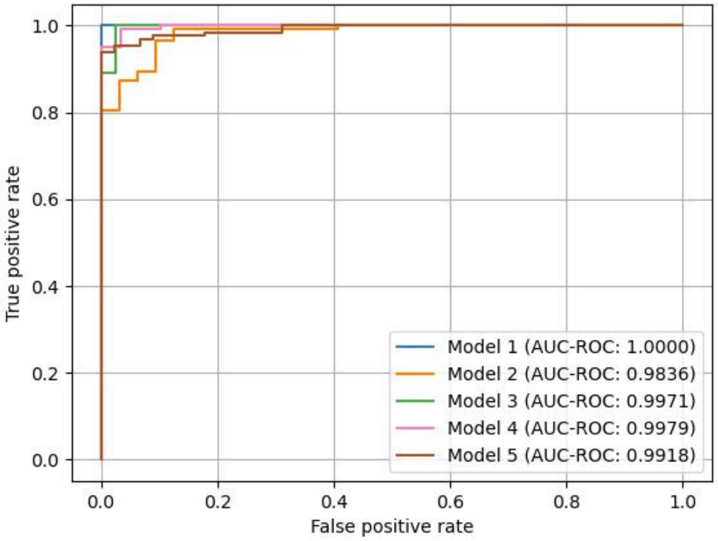


Figure S13: ROC for sex experiment 10%M 90%F (Pneumonia VS COVID-19).

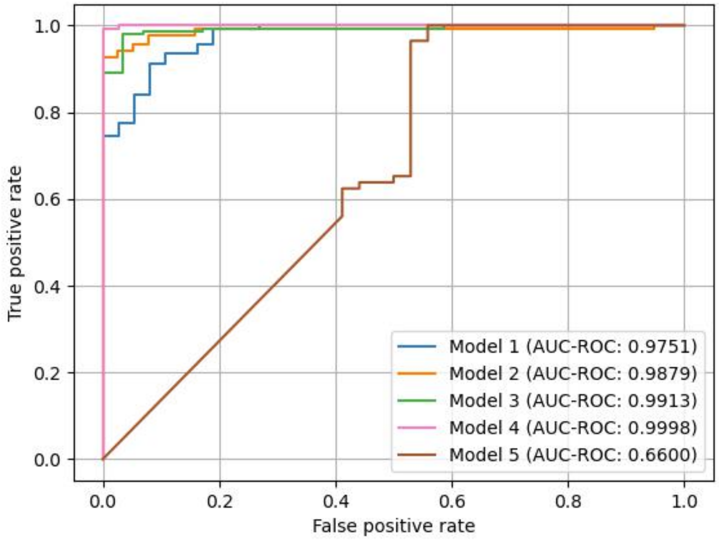


Figure S14: ROC for sex experiment 20%M 80%F (Pneumonia VS COVID-19).

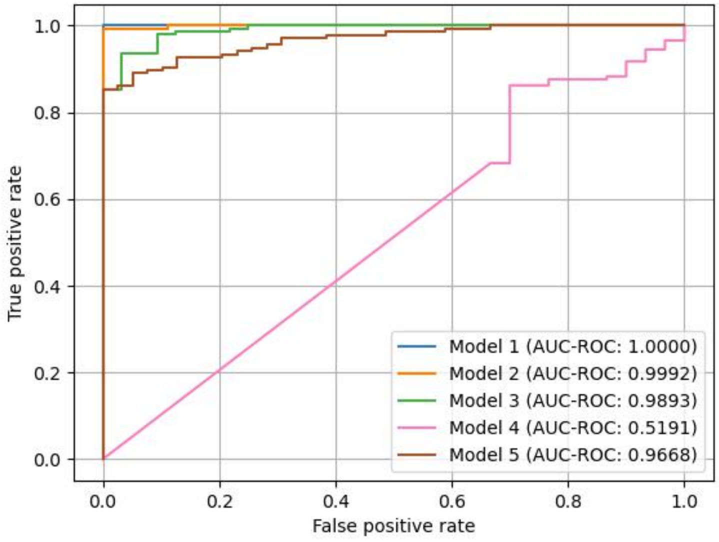


Figure S15: ROC for sex experiment 30%M 70%F (Pneumonia VS COVID-19).

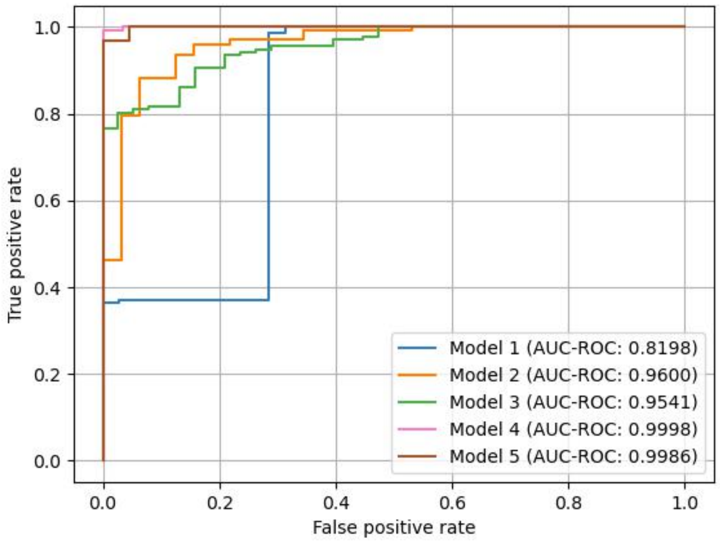


Figure S16: ROC for sex experiment 40%M 60%F (Pneumonia VS COVID-19).

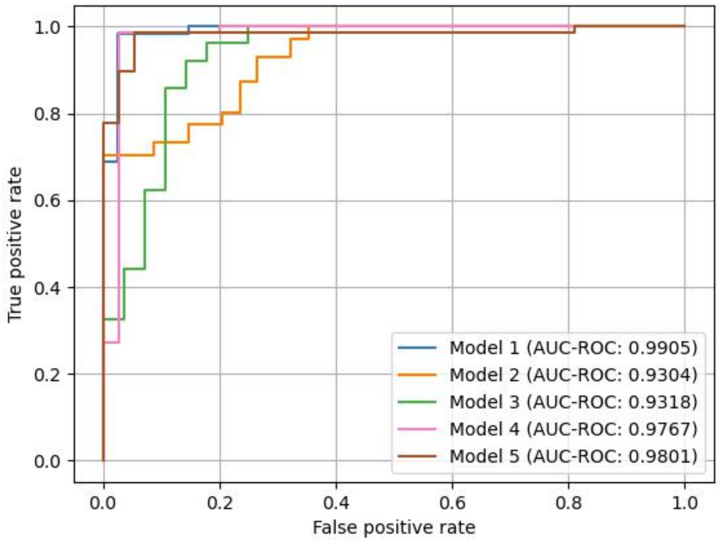


Figure S17: ROC for sex experiment 50%M 50%F (Pneumonia VS COVID-19).

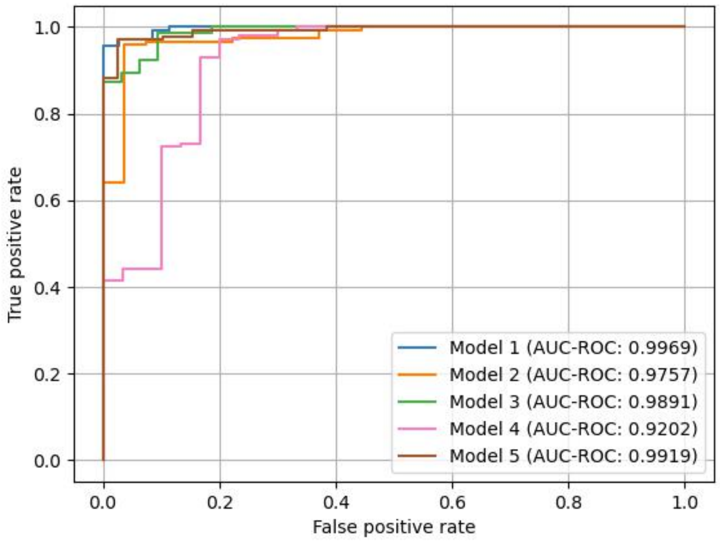


Figure S18: ROC for sex experiment 60%M 40%F (Pneumonia VS COVID-19).

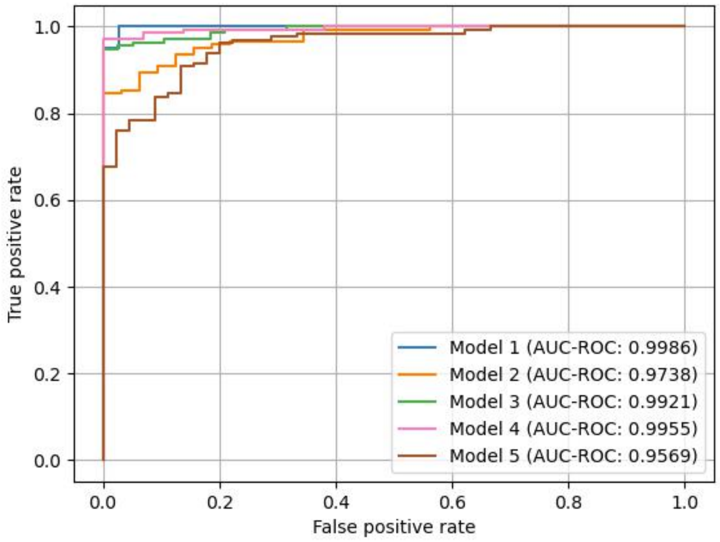


Figure S19: ROC for sex experiment 70%M 30%F (Pneumonia VS COVID-19).

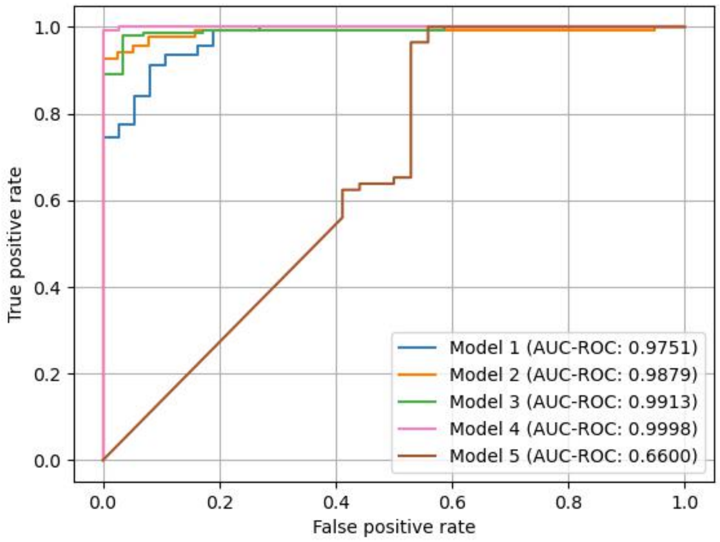


Figure S20: ROC for sex experiment 80%M 20%F (Pneumonia VS COVID-19).

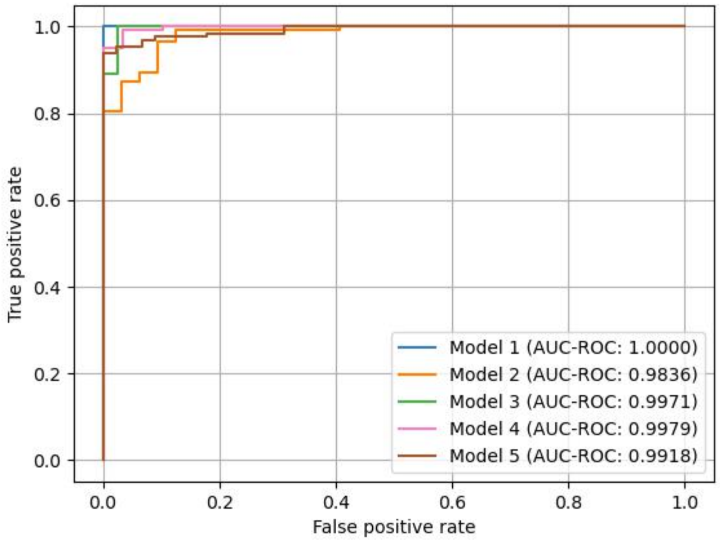


Figure S21: ROC for sex experiment 90%M 10%F (Pneumonia VS COVID-19).

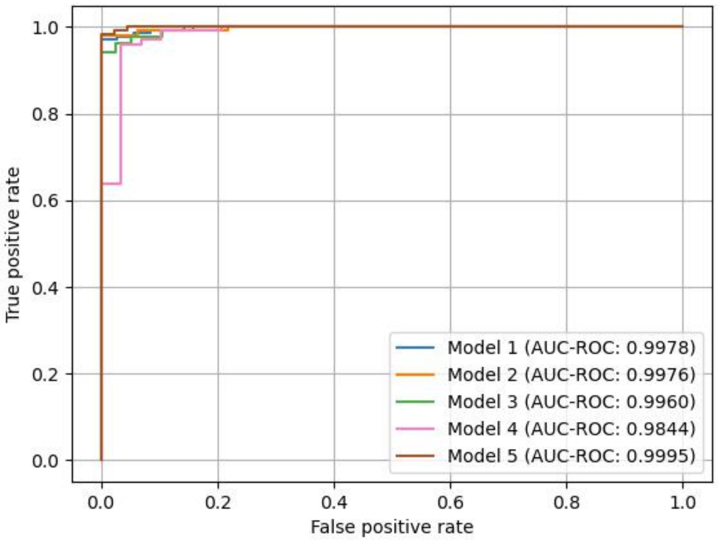


Figure S22: ROC for sex experiment 100%M 0%F (Pneumonia VS COVID-19).

## 1.3 Approach 3: Non-COVID-19 VS COVID-19

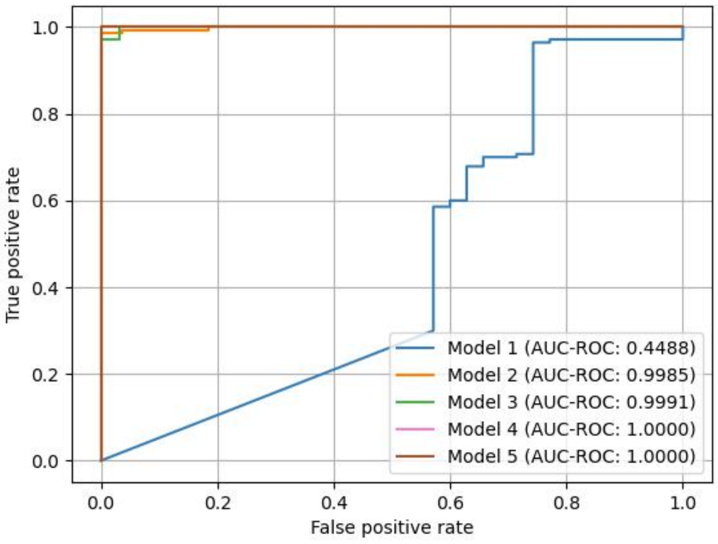


Figure S23: ROC for sex experiment 0%M 100%F (Non-COVID-19 VS COVID-19).

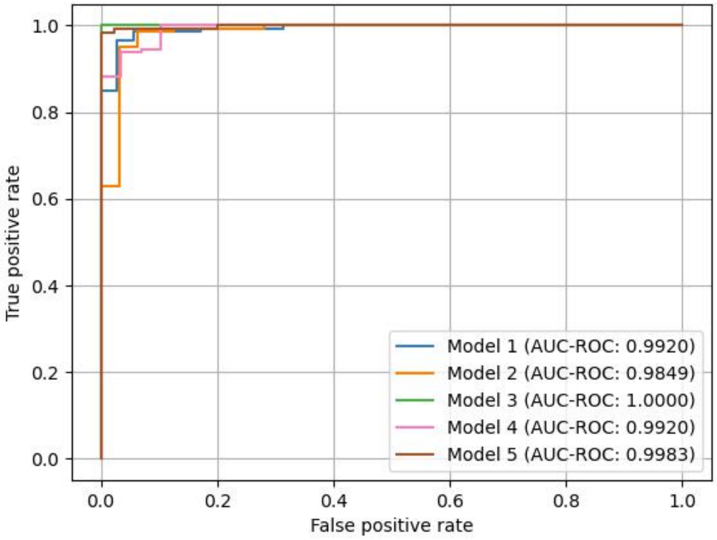


Figure S24: ROC for sex experiment 10%M 90%F (Non-COVID-19 VS COVID-19).

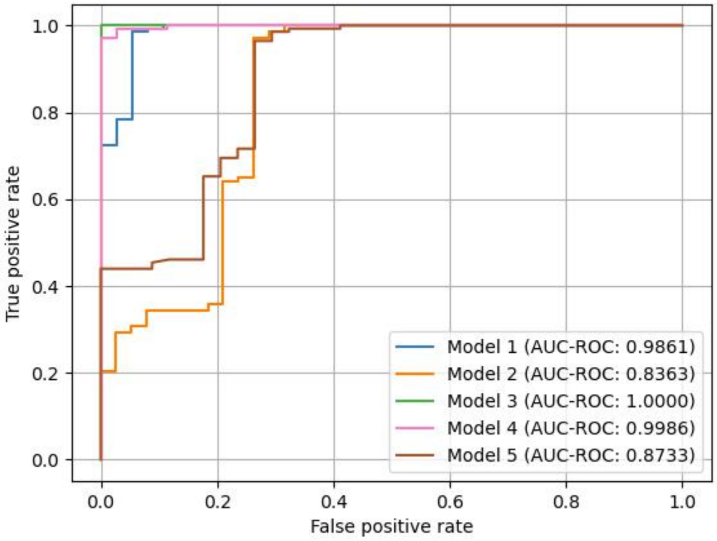


Figure S25: ROC for sex experiment 20%M 80%F (Non-COVID-19 VS COVID-19).

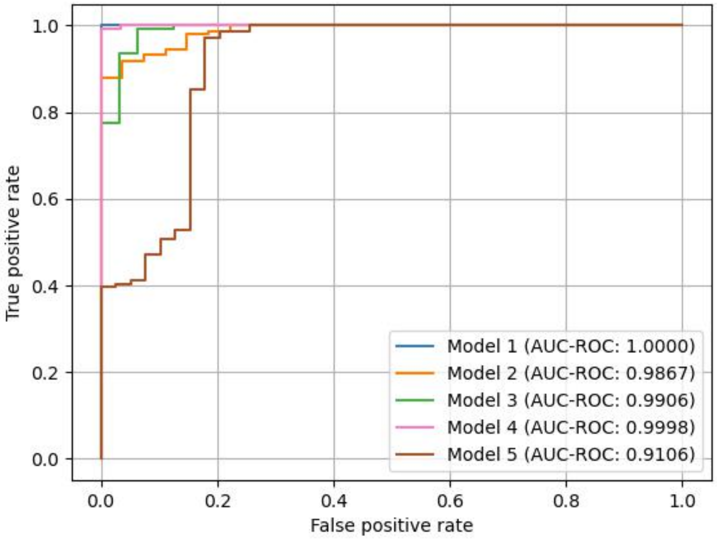


Figure S26: ROC for sex experiment 30%M 70%F (Non-COVID-19 VS COVID-19).

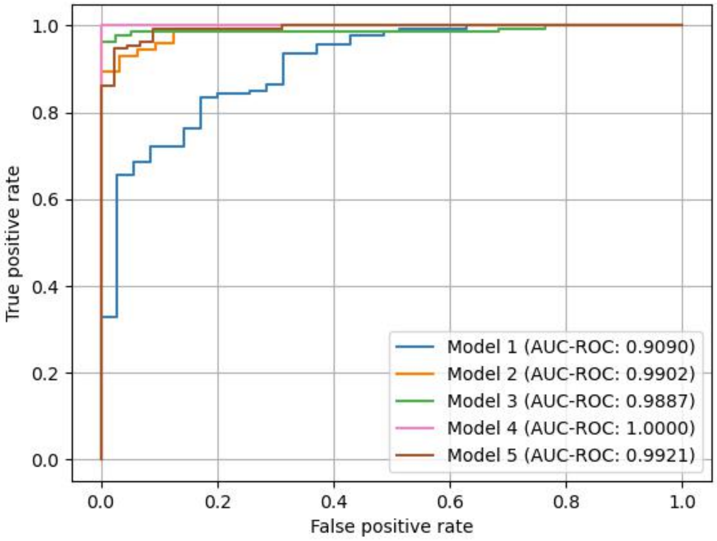


Figure S27: ROC for sex experiment 40%M 60%F (Non-COVID-19 VS COVID-19).

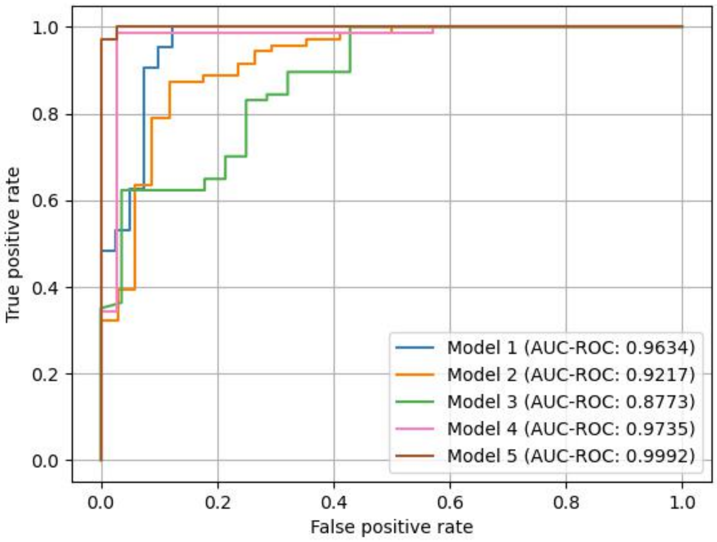


Figure S28: ROC for sex experiment 50%M 50%F (Non-COVID-19 VS COVID-19).

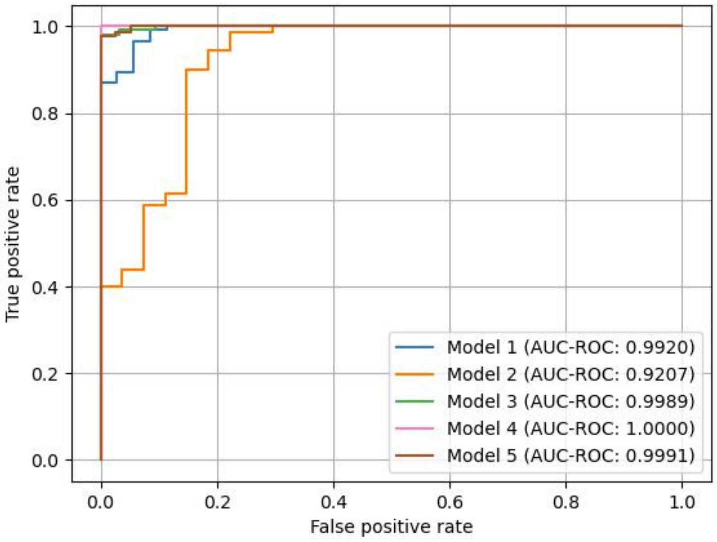


Figure S29: ROC for sex experiment 60%M 40%F (Non-COVID-19 VS COVID-19).

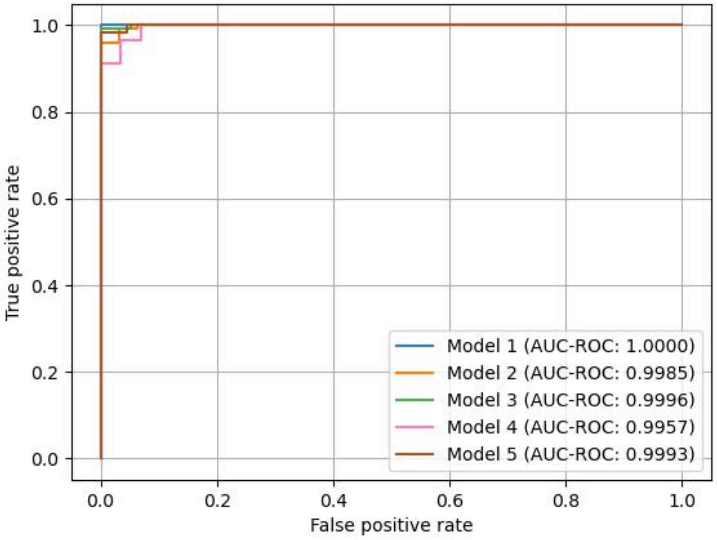


Figure S30: ROC for sex experiment 70%M 30%F (Non-COVID-19 VS COVID-19).

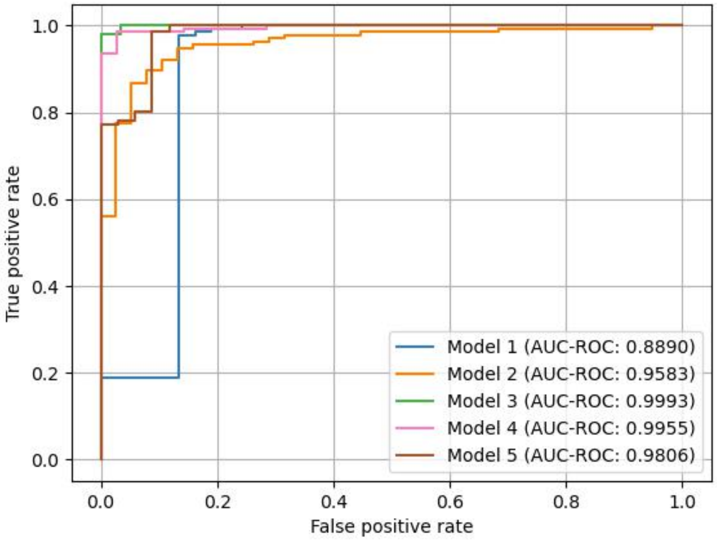


Figure S31: ROC for sex experiment 80%M 20%F (Non-COVID-19 VS COVID-19).

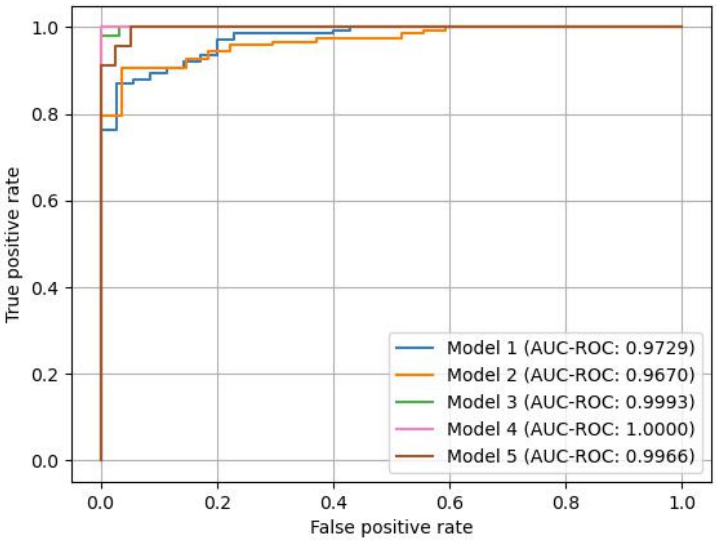


Figure S32: ROC for sex experiment 90%M 10%F (Non-COVID-19 VS COVID-19).

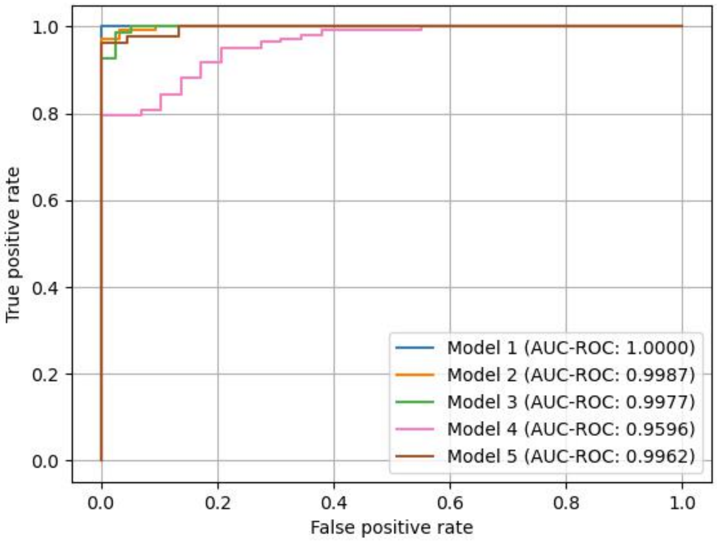


Figure S33: ROC for sex experiment 100%M 0%F (Non-COVID-19 VS COVID-19).

# 2 Age-imbalance analysis

## 2.1 Approach 1: Normal VS COVID-19

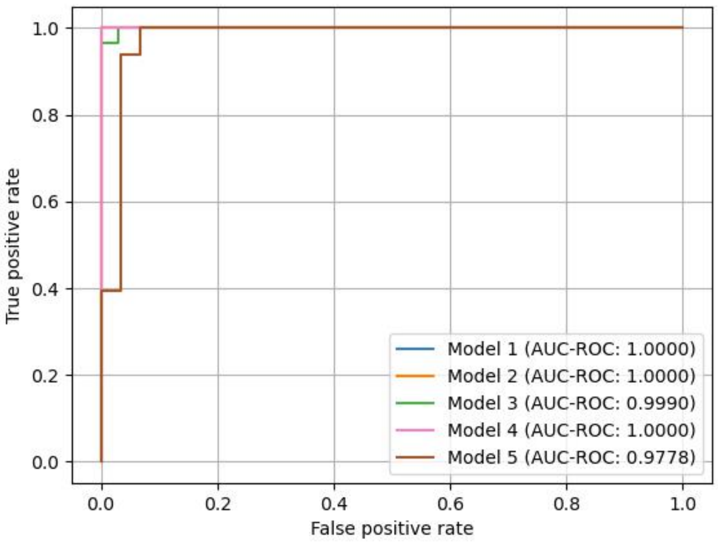


Figure S34: ROC for age experiment with patients from 0 to 40 years old (Normal VS COVID-19).

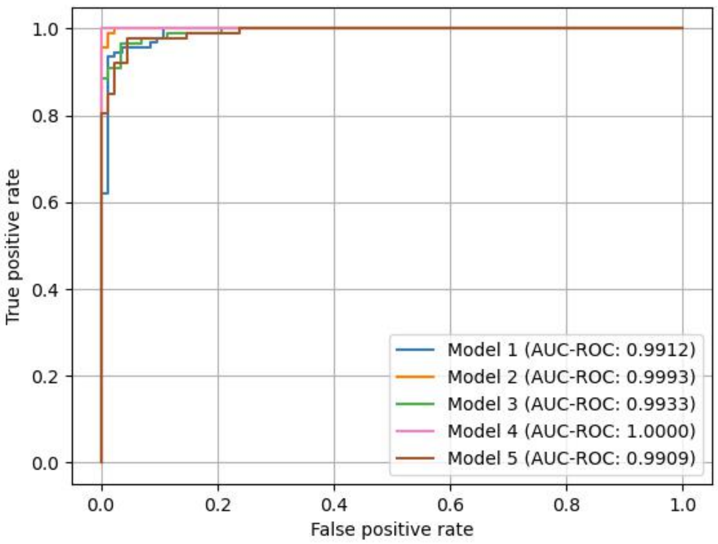


Figure S35: ROC for age experiment with patients from 40 to 50 years old (Normal VS COVID-19).

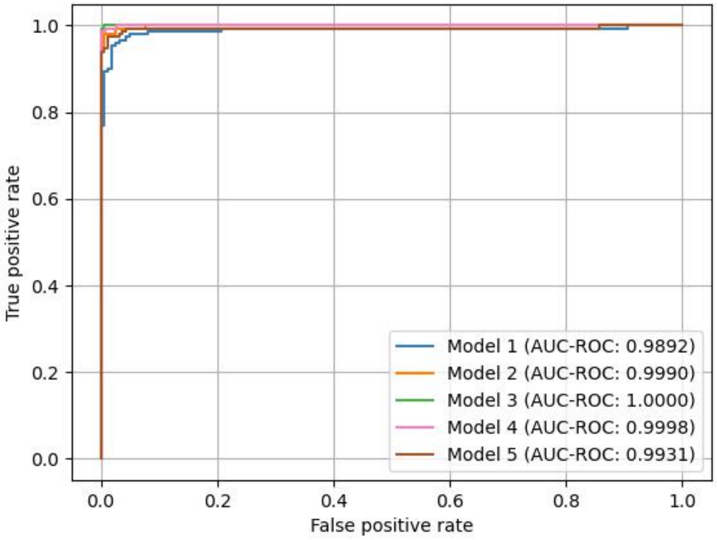


Figure S36: ROC for age experiment with patients from 50 to 60 years old (Normal VS COVID-19).

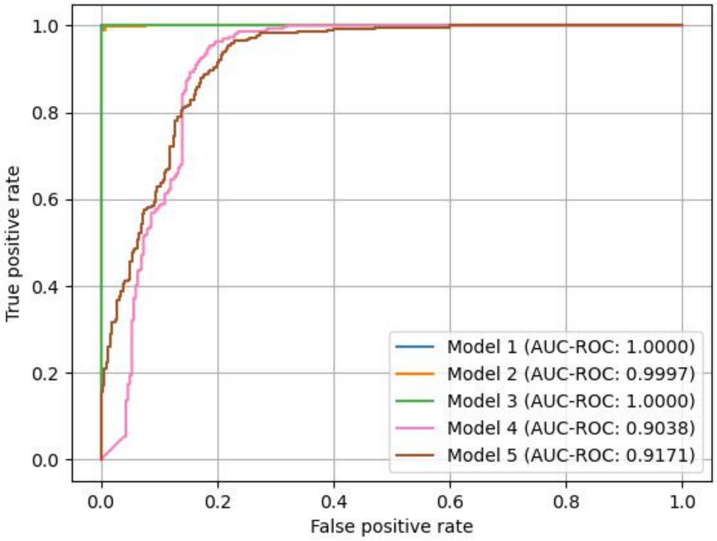


Figure S37: ROC for age experiment with patients from 60 to 70 years old (Normal VS COVID-19).

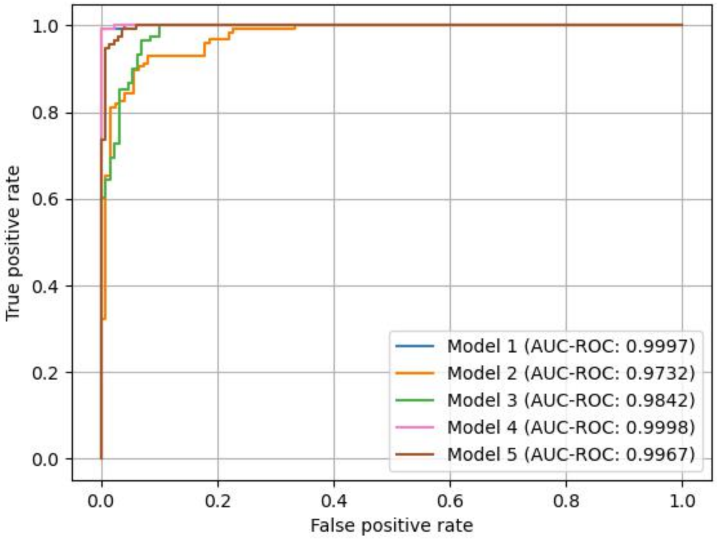


Figure S38: ROC for age experiment with patients from 70 to 80 years old (Normal VS COVID-19).

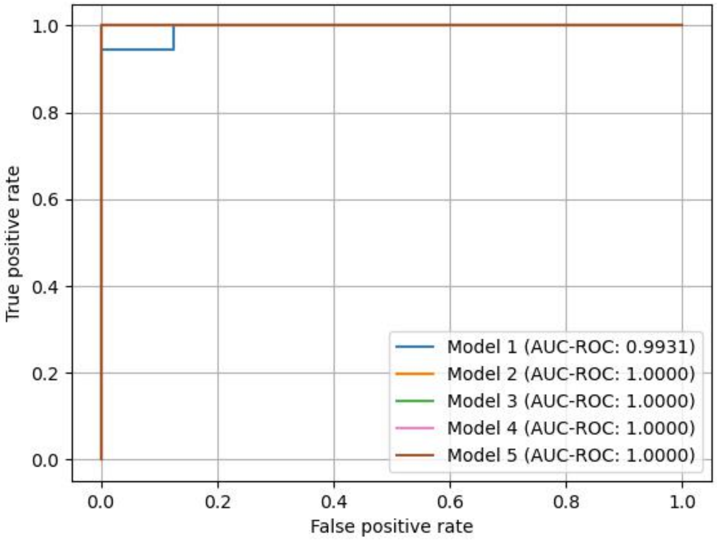


Figure S39: ROC for age experiment with patients from 80 to 100 years old (Normal VS COVID-19).

## 2.2 Approach 2: Pneumonia VS COVID-19

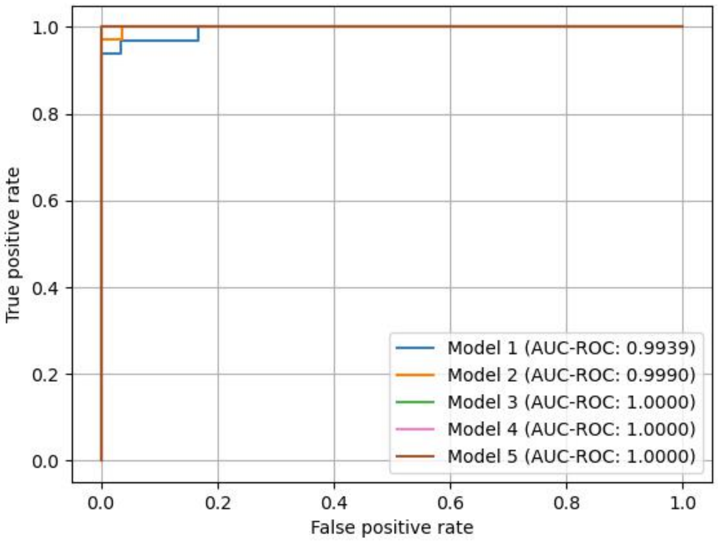


Figure S40: ROC for age experiment with patients from 0 to 40 years old (Pneumonia VS COVID-19).

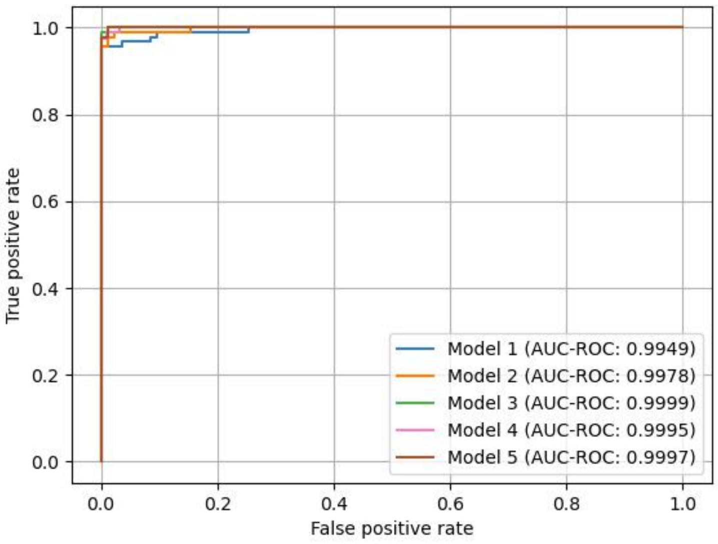


Figure S41: ROC for age experiment with patients from 40 to 50 years old (Pneumonia VS COVID-19).

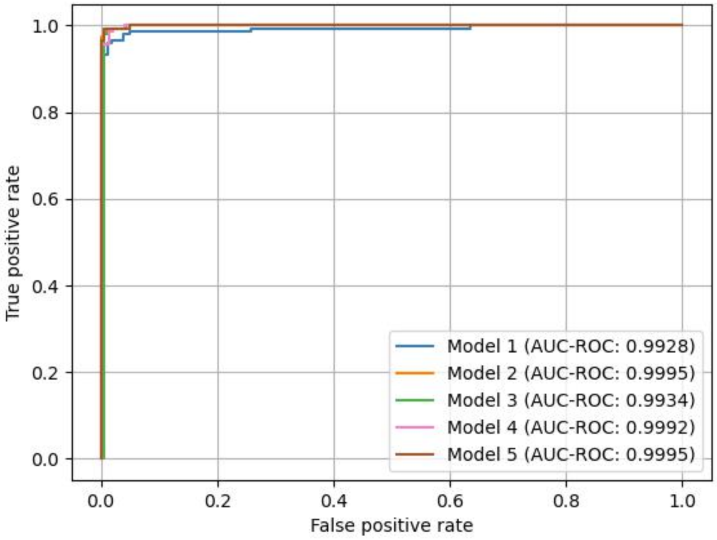


Figure S42: ROC for age experiment with patients from 50 to 60 years old (Pneumonia VS COVID-19).

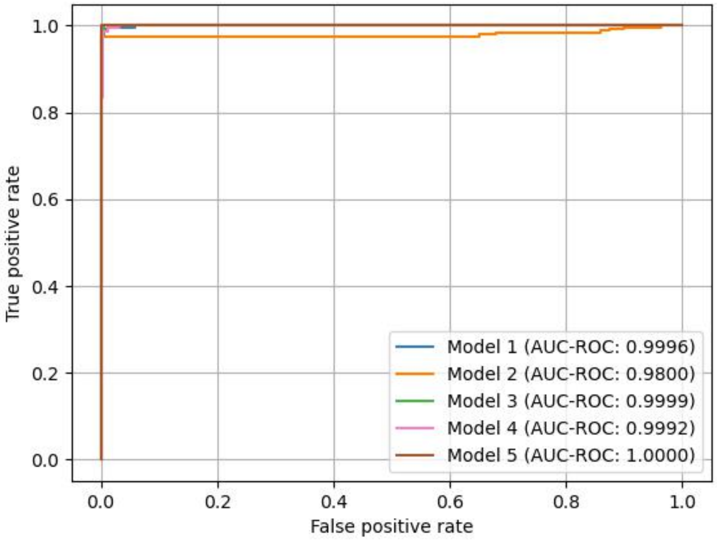


Figure S43: ROC for age experiment with patients from 60 to 70 years old (Pneumonia VS COVID-19).

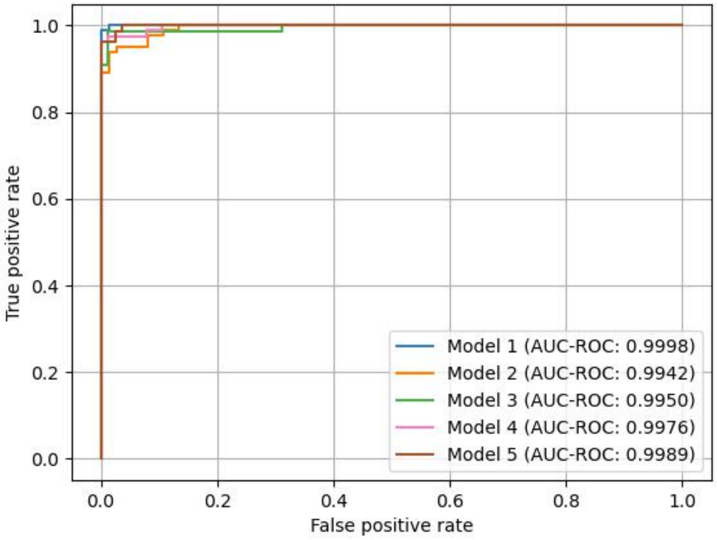


Figure S44: ROC for age experiment with patients from 70 to 80 years old (Pneumonia VS COVID-19).

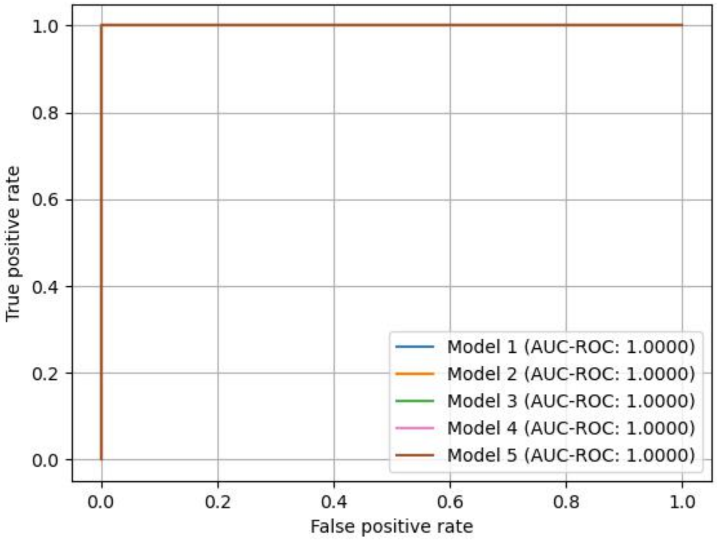


Figure S45: ROC for age experiment with patients from 80 to 100 years old (Pneumonia VS COVID-19).

## 2.3 Approach 3: Non-COVID-19 VS COVID-19

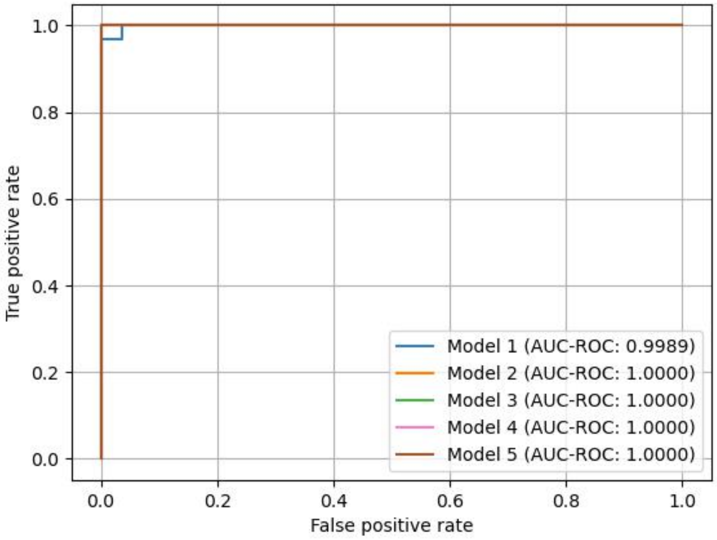


Figure S46: ROC for age experiment with patients from 0 to 40 years old (Non-COVID-19 VS COVID-19).

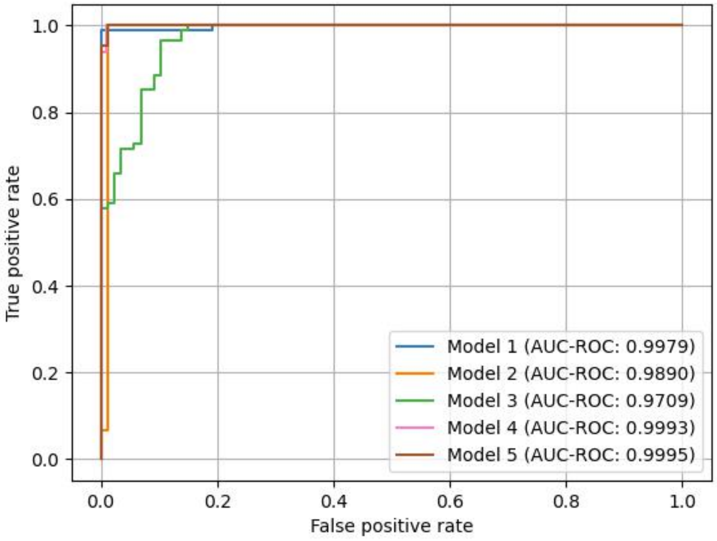


Figure S47: ROC for age experiment with patients from 40 to 50 years old (Non-COVID-19 VS COVID-19).

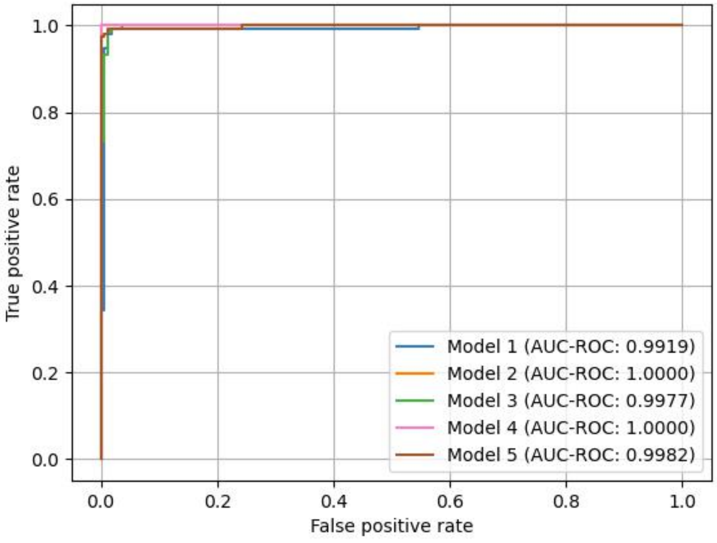


Figure S48: ROC for age experiment with patients from 50 to 60 years old (Non-COVID-19 VS COVID-19).

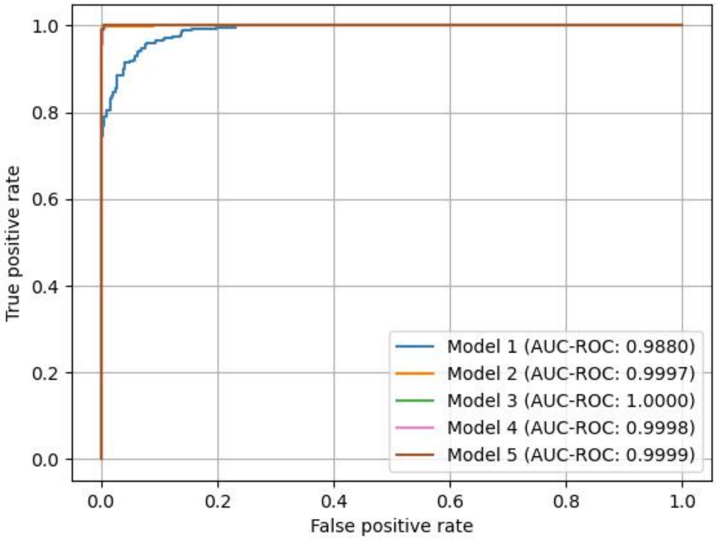


Figure S49: ROC for age experiment with patients from 60 to 70 years old (Non-COVID-19 VS COVID-19).

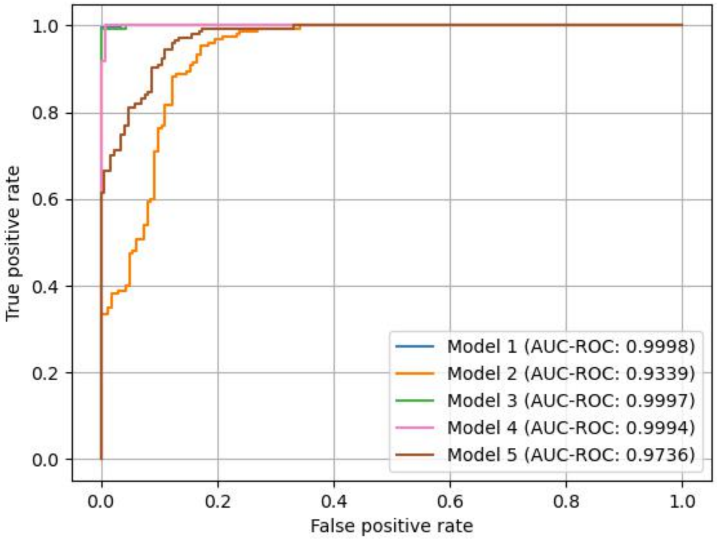


Figure S50: ROC for age experiment with patients from 70 to 80 years old (Non-COVID-19 VS COVID-19).

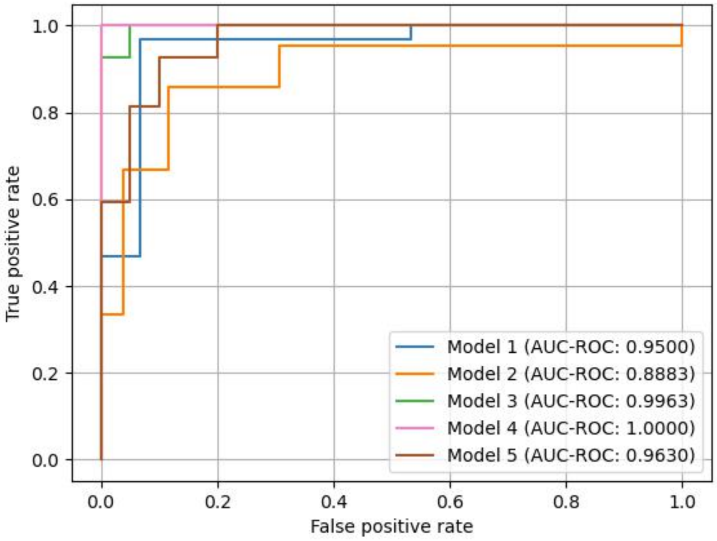


Figure S51: ROC for age experiment with patients from 80 to 100 years old (Non-COVID-19 VS COVID-19).
